# LysM Receptor-Like Kinase and LysM Receptor-Like Protein Families: An Update on Phylogeny and Functional Characterization

**DOI:** 10.3389/fpls.2018.01531

**Published:** 2018-10-24

**Authors:** Luis Buendia, Ariane Girardin, Tongming Wang, Ludovic Cottret, Benoit Lefebvre

**Affiliations:** Laboratoire des Interactions Plantes-Microorganismes, INRA, CNRS, Université de Toulouse, Castanet-Tolosan, France

**Keywords:** defense, symbiosis, MAMP, symbiotic signal, receptor ligand interaction

## Abstract

Members of plant specific families of receptor-like kinases (RLKs) and receptor-like proteins (RLPs), containing 3 extracellular LysMs have been shown to directly bind and/or to be involved in perception of lipo-chitooligosaccharides (LCO), chitooligosaccharides (CO), and peptidoglycan (PGN), three types of GlcNAc-containing molecules produced by microorganisms. These receptors are involved in microorganism perception by plants and can activate different plant responses leading either to symbiosis establishment or to defense responses against pathogens. LysM-RLK/Ps belong to multigenic families. Here, we provide a phylogeny of these families in eight plant species, including dicotyledons and monocotyledons, and we discuss known or putative biological roles of the members in each of the identified phylogenetic groups. We also report and discuss known biochemical properties of the LysM-RLK/Ps.

## Introduction

### Plant Receptor-Like Kinases and Receptor-Like Proteins

**FIGURE 1:**
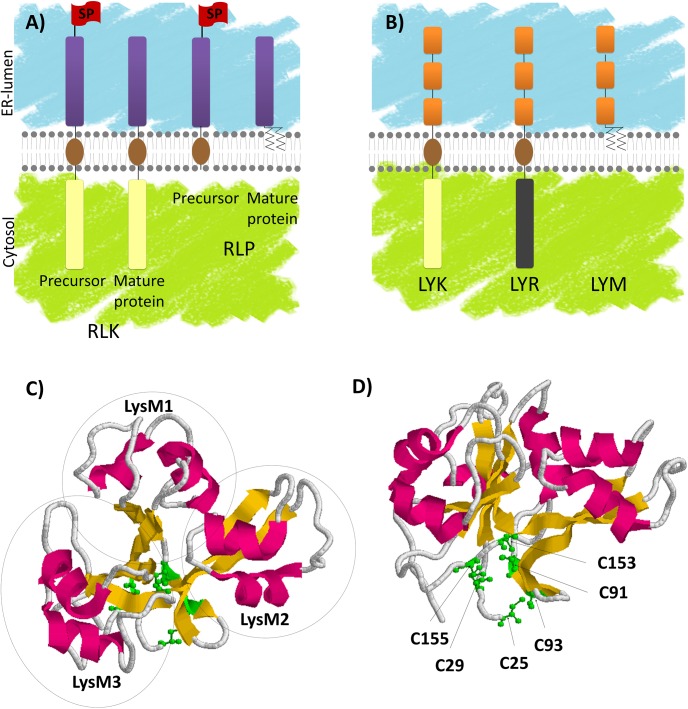
LysM-RLK and LysM-RLP structure, synthesis and maturation. **(A)** Receptor-like kinases (RLKs) are produced by ribosomes associated with the ER. The ECR that is preceded by a SP is translocated into the ER lumen during translation until the TM (brown) is inserted in the lipid bilayer. The ICR is then produced in the cytosol. SPs are cleaved in the ER and the mature proteins are transported through the secretory pathway to their final destinations, mainly the plasma membrane. GPI anchored receptor-like proteins (RLPs) are also produced by ribosomes associated with the ER. After translocation and insertion in the membrane, the SP is cleaved and the ECR is transferred to a GPI anchor. The mature proteins are then transported to their final destinations. **(B)** LysM-RLKs are composed of 3 lysin motifs (LysM, orange) in the ECR, a TM (brown) and an ICR bearing an active kinase (beige, LYK subfamily) or an inactive kinase (gray, LYR subfamily). LysM-RLPs (LYMs) are composed of 3 LysMs in the ECR attached to a GPI anchor. **(C,D)** AtCERK1 3D structure resolved by Liu T. et al. (2012). Images were obtained using the pdb file 4EBY: α-helices are indicated in pink, β-strands are indicated in yellow and C residues are indicated in green. **(C)** Orientation of AtCERK1 ECR highlighting the 3 LysMs (circled) packed together. **(D)** Orientation of AtCERK1 ECR highlighting the C residues involved in disulfide bridges.

Receptor-like kinases (RLKs) are PM proteins found in most eukaryotic organisms. They are transmembrane proteins with an ECR containing a sensor domain, a TM and an ICR containing a domain with homology to protein kinases, involved in signal transduction (Figure 1A). RLKs sense the extracellular environment. They are found in animals, but their number is particularly high in plants in which they have been mainly described to be involved in perception of beneficial or pathogenic microbes (for review, Antolín-Llovera et al., 2012) and in cell/organ communication (for review, Belkhadir et al., 2014; Hazak and Hardtke, 2016). Several RLKs have also been shown to play a role during abiotic stress (for review, Ye et al., 2017). Plant RLKs are divided in subfamilies depending on their ECRs (Shiu and Bleecker, 2003). Among these families, one bears three LysM on the ECR. This subfamily is the main subject of this review.

In their ICRs, plant RLKs have a domain that has homology to the serine/threonine kinases. However, it was shown that in addition to phosphorylation of S/T residues, several plant RLKs can phosphorylate tyrosine residues (Oh et al., 2009; Klaus-Heisen et al., 2011). The canonical form contains a catalytic D residue preceded by an R and is called an RD kinase. A variant kinase domain found in plant RLKs lacks the R preceding the catalytic D and for this reason is called a non-RD kinase. Several non-RD kinases appear to have relatively weak kinase activity *in vitro* compared to the RD kinases, and their kinase activity is partially dispensable for their function (For review, Schwessinger and Ronald, 2012). Kinase domains contain other essential conserved features among which a G-rich loop involved in nucleotide binding. Other variant kinase domains found in plant RLKs lack conserved features such as the G-rich loop and do not exhibit auto-phosphorylation activity *in vitro*. The latter are called dead-kinases or pseudo-kinases.

Another family of plant proteins possess ECRs similar to those of RLKs but lacks ICRs. Among these proteins, called RLPs, some contain only the ECR and are soluble while others are anchored in membrane either with through a TM or a GPI anchor (Figure 1A).

Plant RLKs and GPI-anchored RLPs are mainly found at the PM, although they transiently accumulate in internal compartments of the secretory or endocytic pathways during their life cycle. Indeed, as integral PM proteins, they are produced at the ER. RLKs are type I transmembrane proteins. They bear a SP at their N-terminus (Figure 1A) allowing translocation of the ECR in the ER lumen during protein synthesis. SP is then cleaved, TM is embedded in the ER membrane and the RLKs follow the secretory pathway to the PM. From the PM they can be internalized at the end of their life cycle or after ligand perception through the endocytic pathway and they are ultimately degraded in the lytic vacuole (for review, Beck et al., 2012).

### Lysin Motif Receptor-Like Kinases

Lysin motif receptor-like kinases (LysM-RLKs) and lysin motif receptor-like proteins (LysM-RLPs) are subfamilies of plant RLK/Ps that contain three LysMs in their ECR (Figure 1B). A LysM is a protein domain of about 40 AA found in most living organisms except in Archaea (Buist et al., 2008). Its name originates from its identification in bacterial autolysin proteins that hydrolyze bacterial PGN and lead to cell lysis. Although not highly conserved in terms of primary sequence, LysMs have highly conserved secondary and tertiary structures consisting of two α-helices stacking onto two antiparallel β-sheets as determined by NMR spectroscopy or X-Ray crystallography (Bateman and Bycroft, 2000; Bielnicki et al., 2006; Liu T. et al., 2012; Maxwell et al., 2013; Sánchez-Vallet et al., 2013; Mesnage et al., 2014; Wong et al., 2014; Koharudin et al., 2015; Leo et al., 2015; Liu et al., 2016). Highly conserved C pairs separated by one AA (CXC) are found between the LysMs of all plant LysM-RLK/Ps. These C pairs are involved in disulfide bridges (Lefebvre et al., 2012; Liu T. et al., 2012; Liu et al., 2016) that pack the three LysMs together (Figures 1C,D).

Two main types of plant LysM-RLKs can be defined based on their kinase domains (Figure 1B). The first type, named LYK (Limpens et al., 2003), has a canonical RD kinase and shows *in vitro* autophosphorylation activities (Petutschnig et al., 2010; Klaus-Heisen et al., 2011; Madsen et al., 2011; Zeng et al., 2012). The second type, named LYR (Arrighi et al., 2006), carries an aberrant kinase lacking some conserved features such as the G-rich loop, and does not exhibit either auto-phosphorylation or *trans*-phosphorylation activities when tested *in vitro* (Arrighi et al., 2006; Madsen et al., 2011). GPI-anchored LysM-RLPs are also found in plants and named LYMs (Arrighi et al., 2006).

Most of the LysM-RLK/Ps that have been studied were shown to perceive structurally related GlcNAc containing molecules and/or to be involved in plant-microbe interactions including establishment of defense responses or root endosymbioses. In this review, we report the currently known biological roles and biochemical functions of plant LysM receptor proteins and discuss conservation or evolution of LysM-RLK/P roles and functions in various phylogenetic groups.

### Microbe-Associated Molecular Pattern Triggered Immunity

One layer of plant defense against pathogenic microbes involves perception by plants of conserved microbial signatures also called MAMPs, and consequently induction of MTI. MTI mainly consists in basal defense mechanisms such as cell wall reinforcement, stomatal closure and synthesis of antimicrobial compounds that can lead in some conditions to cell death. Many plant RLKs are involved in MAMP perception and signaling (for review, Schwessinger and Ronald, 2012). Because MAMPs are conserved microbial signatures, they are not specific to pathogens but are also present in beneficial microbes. Specific signatures can also be perceived by plants. In most cases, these specific signatures are proteins called effectors. Effectors are secreted by microbes to manipulate plant signaling, defense or metabolism and the effector repertoire is highly variable within microbial species. Recognition of such proteins produced by pathogens can induce ETI that in most cases leads to cell death.

**FIGURE 2:**
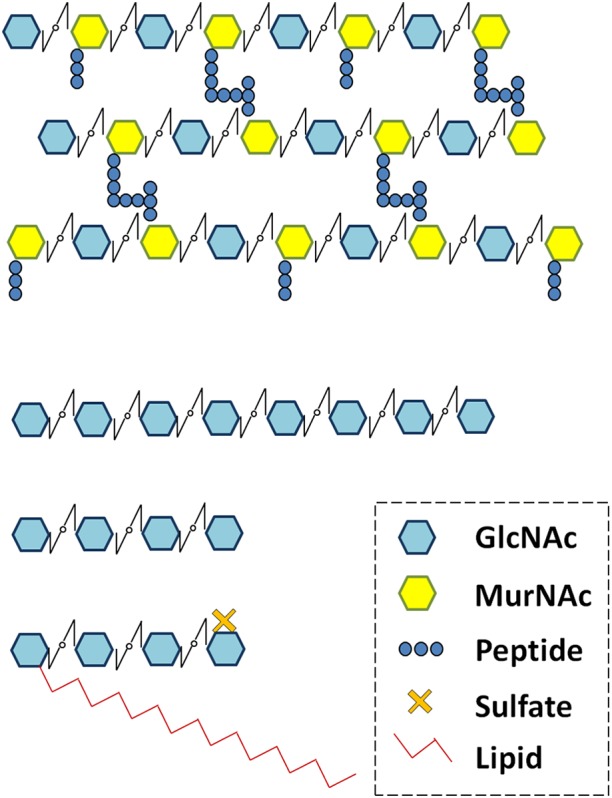
Peptidoglycan (PGN), chitooligosaccharides (CO4 and CO8) and lipo-chitooligosaccharide (LCO-IV) schematic structures. Schematic structures of various *N*-acetyl glucosamine (GlcNAc) containing molecules produced by microorganisms. GlcNAc residues are associated to *N*-acetylmuramic acid (MurNAc) in PGN and to lipid in LCOs.

Plant treatment with various MAMPs typically induces similar responses (such as alkalinisation of the extracellular medium, ROS production, MAP kinase phosphorylation and induction of defense-related gene transcription). These responses have been used to identify and characterize MAMPs. Chitin fragments are typical fungal MAMPs. Chitin is a long-chain β-1,4 GlcNAc polymer, which is the major component of fungal cell walls. Although chitin is insoluble, COs are GlcNAc oligomers (Figure 2), soluble at least up to a degree of polymerization of 8 GlcNAc residues. COs can be produced by chitin cleavage through the action of plant secreted chitinases. Chitin and COs are sometimes used indiscriminately in the literature leading to confusion. For this reason, here we refer to chitin as long insoluble polymers and we mention the degree of polymerization of CO (i.e., CO8 for 8 GlcNAc oligomers). CO8 has been shown to be the most active oligomer among COs for activation of defense-related responses (Kuchitsu et al., 1997). PGN fragments are typical bacterial MAMPs. PGN is a major component of bacterial cell walls. It is a polymer of alternating GlcNAc and *N*-acetylmuramic acid residues, branched with AAs. Like chitin, PGN is insoluble, while muropeptides (Figure 2) are soluble PGN fragments. Chitin and PGN fragments are both perceived by LysM-RLK/Ps as detailed below.

Many other MAMPs do not contain GlcNAc. One of the best characterized bacterial MAMPs is flagellin. Various flagellin peptides are perceived by RLKs in animals and plants (for review, Fliegmann and Felix, 2016). The flg22 peptide (originally identified in *Pseudomonas aeruginosa*; Felix et al., 1999) is perceived in *Arabidopsis thaliana* by AtFLS2, a leucine rich repeat receptor-like kinase (LRR-RLK, Gomez-Gomez and Boller, 2000; Chinchilla et al., 2006). Another well characterized bacterial MAMP is the elf18 peptide found in the bacterial elongation factor (EF-Tu) which is perceived in *A. thaliana* by another LRR-RLK called AtEFR (Zipfel et al., 2006).

### Root Endosymbioses

Plants also interact with many beneficial microbes. AMF can colonize the roots of most terrestrial plants, by establishing an extended hyphal network in the soil and by providing plants with mineral nutrients collected in the soil. Nitrogen fixing bacteria called Rhizobia and Frankia are able to trigger the formation of particular plant root organs called nodules, in phylogenetically related legumes and actinorhizal plants, respectively. Inside nodules, these bacteria can efficiently reduce gaseous atmospheric nitrogen (N_2_) to ammonia (NH_3_) and hence provide a nitrogen source to plants. For this reason, these bacterial genera are extremely important for plant nutrition. Despite the differences in the nature of the microorganisms involved, the AMS and the RNS share commonalities. The mechanism of RNS establishment is considered to originate from the more ancient AMS. Notably, plant genes that control a signaling pathway, called the CSSP, are required for establishment of both AMS and RNS. CSSP activation leads to the production and decoding of oscillations in the calcium concentration (also called calcium spiking) in and around plant cell nuclei. Genes that code for all the components of the CSSP are only found in plants that can establish at least one of these symbioses (Delaux et al., 2014). In such plants, mutations in CSSP genes lead to an absence of AMF penetration at the root epidermis (for review, Gutjahr and Parniske, 2013). In legume and actinorhizal plants, mutations in CSSP genes also block nodule development and bacterial colonization of plant roots (for review, Svistoonoff et al., 2014).

The Nod-factors are well known Rhizobial secreted molecules essential for bacterial recognition by legumes and subsequently for Rhizobial root colonization. Nod-factors are LCOs composed of a core structure of 4 or 5 GlcNAc residues in which the terminal non-reducing sugar is substituted with an acyl chain (Figure 2). In addition, Rhizobial LCOs bear other substitutions that are characteristic of bacterial strains and important for host specificity (Fliegmann and Bono, 2015). AMF also secrete LCOs similar to those produced by Rhizobia (Maillet et al., 2011), as well as short-chain COs (CO4 and CO5, Figure 2, Genre et al., 2013) that might correspond to LCO precursors. Exogenous application of LCOs or short-chain COs activates plant responses such as extracellular medium alkalinization (Staehelin et al., 1994; Felle et al., 1996), calcium spiking (Oldroyd et al., 2001; Harris et al., 2003; Sun et al., 2015), or promotion of lateral root development (Olah et al., 2005; Sun et al., 2015; Herrbach et al., 2017) in various plant species including nodulating and non-nodulating plants. These plant responses have been shown to be CSSP-dependent. In addition, regulation of symbiosis-related gene transcription by treatment with LCOs has been shown in legumes (Combier et al., 2008; Camps et al., 2015; Hohnjec et al., 2015) but not yet reported in non-legumes. The CO4 and CO5 produced by AMF are referred as Myc-COs or short-chain COs and are currently considered to play a role in AMS establishment in contrast to the long-chain COs, CO7 and CO8 described to be defense elicitors. Effectors are also produced by AMF (Kloppholz et al., 2011; Kamel et al., 2017) and some rhizobia (Deakin and Broughton, 2009; Yang et al., 2010; Okazaki et al., 2013), and can be involved in symbiosis establishment.

## Phylogenetic ANALYSIS

### Methodology

Studies that deal with functional characterization of LysM-RLK/Ps have been performed only within a few species that represent the genetic diversity of higher plants. This includes dicotyledons (*A. thaliana*, *Medicago truncatula, Lotus japonicus*, and *Solanum lycopersicum*) and a monocotyledon (*Oryza sativa*). Although several LysM-RLK/P phylogenetic trees have been published (Arrighi et al., 2006; Zhang et al., 2009; Lohmann et al., 2010; Shimizu et al., 2010; Zeng et al., 2012; Buendia et al., 2016; Bono et al., 2018) none of these phylogenies include all the listed species at the same time. Moreover naming of LysM-RLK/Ps has been done independently in each species making the comparison between species complicated.

**FIGURE 3:**
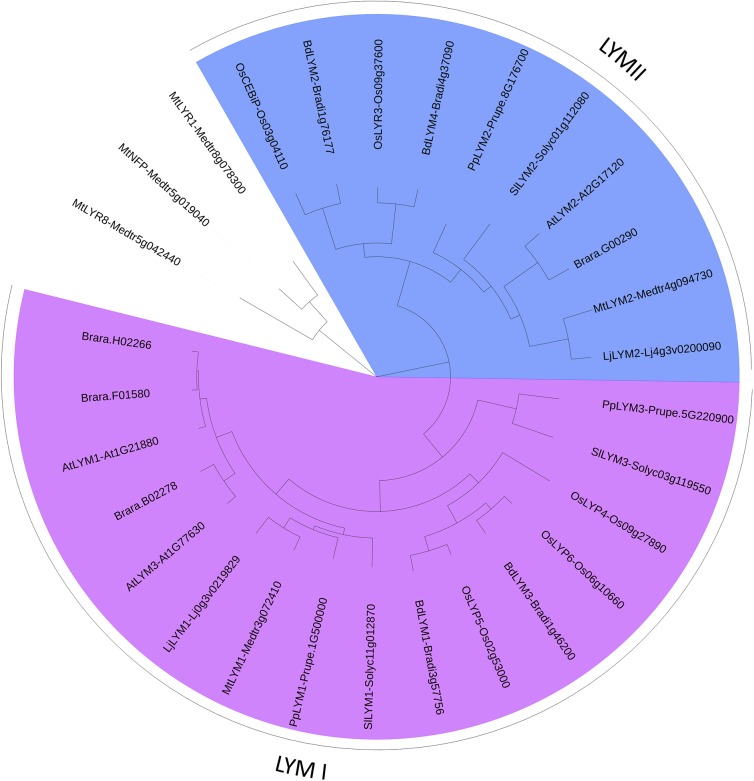
PhyML phylogenetic tree of the LYMs. Different phylogenetic groups are shown in different colors. ECRs of 3 LYR proteins were used as outgroup sequences. The sequences corresponding to the LYM proteins were aligned with Mafft (v7.271; Katoh et al., 2002) with the following parameters: maxiterate = 1000, retree = 1, genafpair = true. The best evolutionary model fitting the alignment was identified using ProtTest (v2.4). This best model was: WAG + I + G, alpha = 2.69, p-inv = 0.05. The phylogenetic tree was computed using a maximum-likelihood method with phyml (v20130805; Guindon et al., 2010). The branch confidence was evaluated using the Approximate Likelihood-Ratio Test (Anisimova and Gascuel, 2006). Finally, the tree was drawn with Itol v3 (Letunic and Bork, 2016).

**FIGURE 4:**
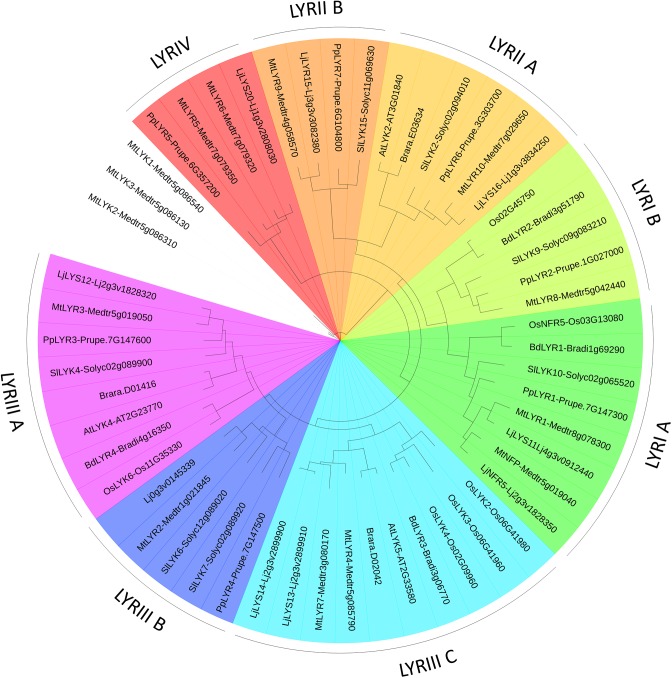
PhyML phylogenetic tree of the LYRs. Different phylogenetic groups are shown in different colors. Three LYK proteins were used as outgroup sequences. The same protocol as for the LYM family tree (Figure 3) was used, except that the best model fitting the alignment was LG + I + G, alpha = 1.71, p-inv = 0.06.

To discuss the evolution of LysM-RLK/Ps in higher plants, we have inferred phylogenetic trees using phyML (Figures 3, 4, 6) and MrBayes (Supplementary Figures S1–S4). In addition to the species mentioned above, we used the sequences of two additional dicotyledons (*Prunus persica* and *Brassica rapa*) and one more monocotyledon (*Brachypodium distachyon*) in which the genome sequences have been published. We performed manual correction of many gene structure predictions (see notes in Supplementary Table S1). Phylogenetic trees were inferred independently with predicted protein sequences of the LYMs (Figure 3 and Supplementary Figure S1), LYRs (Figure 4 and Supplementary Figure S2) or LYKs (Figure 6 and Supplementary Figure S3). Some proteins identified as putative LysM-RLK/Ps were not used for the phylogenetic analysis because they were truncated or their sequence/existence was uncertain (these proteins are indicated in italics in Supplementary Table S1). We focused our analysis on the membrane-anchored LysM-RLPs (LYMs) and we did not consider the soluble LysM-RLPs containing 3 LysMs (called LYP clade II in Zhang et al., 2009). We used PredGPI^1^ to identify the GPI anchor sites in the LYMs. Orthologous genes based on the phylogenetic trees are arranged in Supplementary Table S1 in lanes with a color code. To reinforce ortholog identification, we reported the intron–exon structure in Supplementary Table S1 which is almost conserved among all the orthologs. Minor differences might be due to evolutionary changes or to residual errors in gene structure predictions.

**FIGURE 5:**
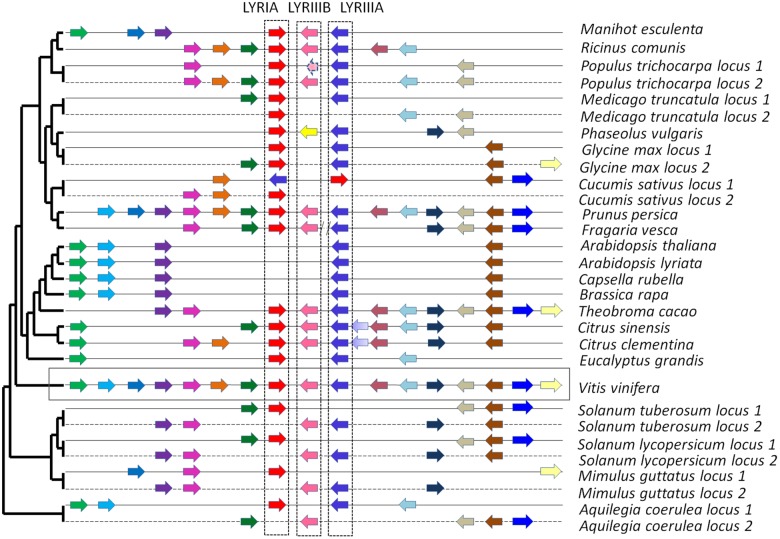
Syntenic localization of the members of the phylogenetic groups LYRIA (red), LYRIIIB (pink) and LYRIIIA (blue). The orthologs are represented by similar color in the various genomes. Synteny was built by using the genome of *Vitis vinifera* as reference. On the left, the phylogenetic tree of the species is that of Phytozome v10.

**FIGURE 6:**
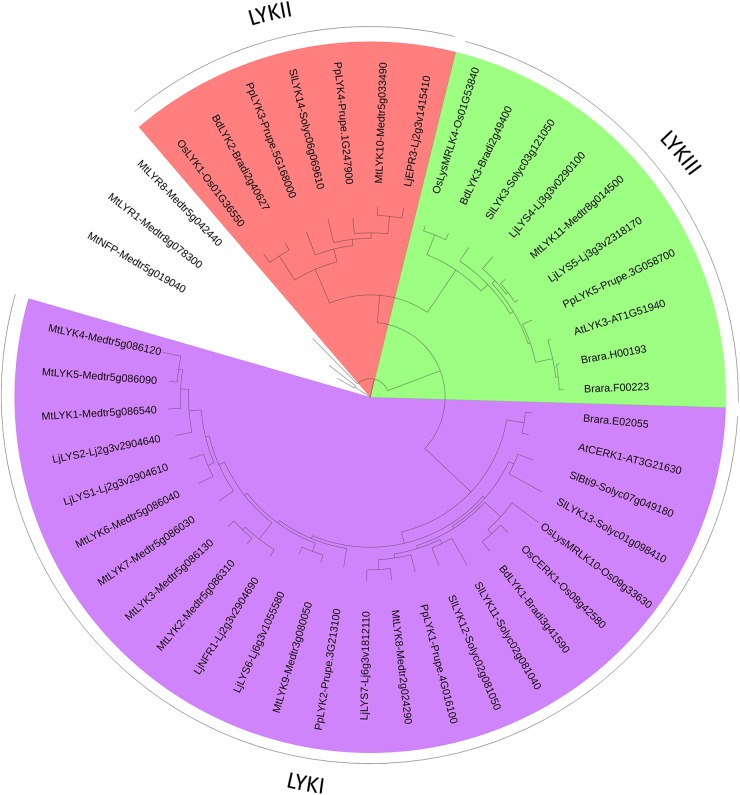
PhyML phylogenetic tree of the LYKs. Different phylogenetic groups are shown in different colors. Three LYR proteins were used as outgroup sequences. The same protocol as for the LYM family tree (Figure 3) was used, except that the best model fitting the alignment was JTT + I + G, alpha = 1.32, p-inv = 0.1.

We found a high variability in the number of LysM-RLKs (5–22) and membrane-anchored LysM-RLPs (2–5) between the eight plant species analyzed, with an expansion of the LysM-RLKs in dicotyledons (except in the Brassicaceae) versus monocotyledons and an expansion of membrane-anchored LysM-RLPs in monocotyledons versus dicotyledons. Legumes showed the highest number of LysM-RLKs and the lowest number of membrane-anchored LysM-RLPs. Although the number of genes is highly variable, phylogenetic groups can be distinguished with members in almost all species. We distinguished 2 phylogenetic groups of LYMs (Figure 3 and Table 1), 2 phylogenetic groups of LYRs (Figure 4), and 3 phylogenetic groups of LYKs (Figure 6) common to dicotyledons and monocotyledons. Two additional phylogenetic groups of LYRs and several subgroups were found only in dicotyledons. We propose to name the phylogenetic groups in the trees as LYM, LYR, and LYK with one number, and a letter when subgroups can be distinguished (Supplementary Table S1). Below and in Supplementary Table S1, we also reported the nomenclature proposed by Zhang et al. (2009). Most phylogenetic subgroups have one member in all species with few exceptions of duplications in particular species. Two phylogenetic groups (LYRI and LYKI) have, however, encountered many duplication events that explain most of the variability in the number of LysM-RLKs between species.

**Table 1:** LysM-RLPs belonging to the phylogenetic groups LYMI or LYMII found in the 8 species analyzed.

	*M. truncatula*	*L. japonicus*	*P. persica*	*A. thaliana*	*B. rapa*	*S. lycopersicum*	*B. distachyon*	*O. sativa*
LYMI	Medtr3g072410	Lj0g3v0219829	Prupe.1G500000	At1g21880	Brara.F01580	Solyc11g012870	Bradi3g57756	Os06g10660
	LYM1		PpLYM1	AtLYM1		SlLYM1	BdLYM1	OsLYP6
					Brara.H02266		Bradi1g46200	Os02g53000
							BdLYM3	OsLYP5
				At1g77630	Brara.B02278			Os09g27890
				AtLYM3				OsLYP4
			Prupe.5G220900			Solyc03g119550		
			PpLYM3			SlLYM3		
LYMII	Medtr4g094730	Lj4g3v0200090	Prupe.8G176700	At2g17120	Brara.G00290	Solyc01g112080	Bradi1g76177	Os03g04110
	LYM2		PpLYM2	AtLYM2		SlLYM2	BdLYM2	OsCEBiP
							Bradi4g37090	Os09g37600
							BdLYM4	OsLYP3

Almost no introns were found in the 5′ part of the genes encoding the 3 LysMs, in either LYRs, LYKs, or LYMs. In contrast, strong differences of intron number were observed in the sequence encoding the extracellular juxta membrane region and the ICR (0/1 in LYRs, 3/4 in LYMs and 9/12 in LYKs). This suggests independent combinations of the sequence encoding the 3 LysMs with either one encoding a dead kinase for the LYR ancestor, one encoding an active kinase for the LYK ancestor and one encoding a site for GPI anchoring for the LYM ancestor. This might have happened at an early time of plant evolution. Indeed, members of the phylogenetic groups LYRI and LYKI can be found in more ancient plant genera (Zhang et al., 2009) such as Physcomitrella (bryophytes) and Selaginella (lycophytes).

Biological roles and biochemical functions, as well as the evolution of the number of members for each phylogenetic group are discussed below.

### Description of the Phylogenetic Groups

#### LysM-RLP LYMI (LYP Clade I in Zhang et al., 2009)

One to three members can be found in the phylogenetic group LYMI (Figure 3, Table 1, and Supplementary Figure S1). Studies concerning members of this phylogenetic group have only been performed in *A. thaliana* and *O. sativa*. They are involved in perception of PGN and resistance to bacterial pathogens.

In *A. thaliana*, the two members of this phylogenetic group, *AtLYM1* (*At1g21880*) and *AtLYM3* (*At1g77630*) are required for activation of PGN signaling (Willmann et al., 2011). In *Atlym1* or *Atlym3* knock-out mutants, transcriptional responses to PGN are abolished and growth of the pathogenic bacterial strain *Pseudomonas syringae* pv. tomato DC3000 is increased. The double mutant has a similar phenotype to single mutants suggesting a cooperative role of the two proteins rather than a redundant role. These two genes do not play a role in chitin perception. To demonstrate PGN binding, AtLYM1 and AtLYM3 ECRs were produced in *Escherichia coli*, purified and pulled-down using insoluble PGN. Attachment of AtLYM1 or AtLYM3 ECR to insoluble PGN decreased in the presence of soluble PGN fragments (PGN hydrolyzed by sonication), but not in the presence of CO6, CO8, or LCOs, showing that AtLYM1 and AtLYM3 specifically bind PGN fragments (Willmann et al., 2011). The affinity of AtLYM1 and AtLYM3 for PGN is, however, not known.

In *O. sativa*, *OsLYP4* (*Os09g27890*) and *OsLYP6* (*Os06g10660*), two of the three members in the phylogenetic group LYMI, have been reported to play a similar role in PGN recognition but surprisingly also in CO recognition (Liu B. et al., 2012). In plants with decreased expression of *OsLYP4* or *OsLYP6* by RNAi, responses to PGNs and to CO6 (transcriptional responses and callose deposition) were decreased and lesions due to bacterial (*Xanthomonas oryzae* or *X. oryzicola*) or fungal (*Magnaporthe oryzae*) pathogens were increased. In contrast, overexpression of the two proteins led to decreased lesions in presence of these pathogens. OsLYP4 and OsLYP6 ECRs were also produced in *E. coli*, purified and pulled-down using insoluble PGN or insoluble chitin. Competition assays were performed using CO6 or soluble PGN fragments (PGN hydrolyzed by lysostaphin). Each of these molecules was able to inhibit OsLYP4 and OsLYP6 pull-down either by insoluble chitin or PGN, showing that the same binding site was responsible for CO and PGN fragment perception. The affinity of OsLYP4 and OsLYP6 for PGN or chitin is, however, not known. *OsLYP5* (*Os02g53000*) is also a member of this phylogenetic group and OsLYP5 and OsLYP6 are close paralogs while OsLYP4 is slightly divergent (Figure 3, Liu B. et al., 2012). In contrast to what is mentioned in Liu B. et al. (2012), we found a predicted TM and GPI anchor site in OsLYP5. Using siRNA-Finder (Si-Fi^2^) we found that the construct used to silence *OsLYP6* in Liu B. et al. (2012) is predicted to target *OsLYP5* as well. Thus an analysis of *OsLYP5* role in PGN and chitin perception and an analysis of cross silencing of *OsLYP5* by *OsLYP6* hairpin constructs are required to clarify the differences in the biological role between *A. thaliana* and *O. sativa* orthologs.

#### LysM-RLP LYMII (LYP Clade III in Zhang et al., 2009)

In dicotyledons, only one member was found in this phylogenetic group, while two members were present in monocotyledons (Figure 3, Table 1, and Supplementary Figure S1). Members of this phylogenetic group and especially rice OsCEBIP are among the best characterized LysM-RLK/Ps. They are involved in long-chain CO perception and resistance to fungal pathogens.

In rice, knock-down of Chitin Elicitor Binding Protein (*OsCEBiP*, *Os09g37600*) by RNAi resulted in a decrease of the CO8-induced oxidative burst in a rice cell culture (Kaku et al., 2006) and in an increase of *M. oryzae* colonization in rice plants (Kishimoto et al., 2010). On the contrary, *OsCEBiP* overexpression led to a decrease of lesion size by *M. oryzae.* Its role as a main actor in CO8 perception was further confirmed in cell cultures derived from knock-out plants (Kouzai et al., 2014b). Although OsCEBIP was firstly described to have 2 LysMs, elucidation of its 3D structure (Liu et al., 2016) unambiguously demonstrated that it bears 3 LysMs as all LysM-RLK/Ps. OsCEBiP was originally purified from rice cell cultures (Kaku et al., 2006) in which a binding site for CO8 had been characterized using a radiolabeled CO8 derivative with *Kd* of 5.4 or 29 nM in microsomal fraction (Shibuya et al., 1993) or PM fraction (Shibuya et al., 1996), respectively. Similarly, half maximal incorporation of a radiolabeled photoactivatable CO8 derivative in microsomal fractions of a rice cell culture was about 50 nM (Ito et al., 1997). Using a biotinylated CO8 derivative that can be crosslinked to proteins and detected by Western blotting (with antibodies raised against biotin), it has been shown that the CO8 binding site detected in rice cell culture disappears when *OsCEBIP* is silenced (Shinya et al., 2010) or knocked-out (Kouzai et al., 2014b). OsCEBIP was also expressed heterologously in a tobacco BY-2 cell culture and half saturation was found around 100 nM using the biotinylated CO8 derivative (Shinya et al., 2012). As shown for the CO8 binding site in rice cell cultures (Shibuya et al., 1996), competition assays with different lengths of CO on OsCEBIP expressed in BY-2 cells demonstrated higher affinity for CO8 than for shorter COs (Shinya et al., 2010, 2012). Note that all CO8 derivatives used in these studies have an opened GlcNAc at the reducing-end and were shown to have biological activities lower than CO8 and comparable to CO7. However, this modification does not affect the affinity deduced from the competition assays with unmodified COs. More recently, OsCEBIP ECR was expressed in insect cells and purified. Affinities of 3 μM for CO4 and 4 μM for CO8 were determined by ITC (Liu et al., 2016). The CO binding site was found on the second LysM both by NMR spectroscopy and modeling (Hayafune et al., 2014) and by X-Ray crystallography (Liu et al., 2016). By mutating I150 in LysM2 [named 122 in Hayafune et al. (2014) as numbering started after the SP], it was demonstrated that this residue is critical for CO binding (Hayafune et al., 2014; Liu et al., 2016). Because the binding site on the second LysM is occupied by a CO3 (Hayafune et al., 2014; Liu et al., 2016), it has been hypothesized that CO8 binding occurs through dimerization of OsCEBIP (Hayafune et al., 2014; Liu et al., 2016). CO8 was actually found to induce *in vitro* dimerization of the OsCEBIP ECR produced in *E. coli* (Hayafune et al., 2014), but not of the OsCEBIP ECR produced in insect cells (Liu et al., 2016). It has been also shown that OsCEBIP ECR is able to form homodimers in a yeast two hybrid system and that part of the OsCEBIP is found at a size corresponding to homodimer *in vivo* in absence of COs using blue native polyacrylamide gel electrophoresis and immunodetection (Shimizu et al., 2010). The requirement of dimerization to form a high affinity binding site might explain the low affinity for CO8 found in the OsCEBIP ECR produced in insect cells (Liu et al., 2016) and its inability to discriminate CO4 and CO8 (Liu et al., 2016), in contrast to the previous OsCEBIP biochemical characterization. Interestingly 100 nM of (GlcNβ1,4GlcNAc)_4_, an oligosaccharide alternating *N*-acetylated and non-*N*-acetylated glucosamine (therefore carrying *N*-acetyl moieties only on one face of the polymer) was shown to inhibit CO8-induced OsCEBIP *in vitro* dimerization and ROS production in rice cells (Hayafune et al., 2014). In contrast, 100 nM CO4 did not compete CO8 for these responses (Hayafune et al., 2014). This suggests that two OsCEBIP monomers bind a single CO8 molecule, with each OsCEBIP monomer binding to an opposite face and side of the CO8 molecule.

In wheat and barley, orthologs of OsCEBIP were also shown to be involved in defense against pathogens. Wheat lines that were knockdown for *TaCEBIP* by VIGS showed disease symptoms produced by the fungal pathogen *Mycosphaerella graminicola* line (mutated for an effector involved in virulence) which was reported not to be pathogenic on WT wheat plants (Lee et al., 2014). Barley lines knock-down for *HvCEBIP* by VIGS also showed increased lesions due to *M. oryzae* (Tanaka et al., 2010). In WT lines of these plant species, CO8 binding sites similar to those of rice were detected (Okada et al., 2002) although the corresponding proteins have not been characterized.

In *A. thaliana*, the only member of the phylogenetic group LYMII, AtLYM2 (*At2g17120*) is also a chitin-binding protein. Expressed in BY-2 cells, AtLYM2 showed binding to COs as OsCEBIP (Shinya et al., 2012). Surprisingly, AtLYM2 is not required for general responses to COs (Shinya et al., 2012; Narusaka et al., 2013). However, AtLYM2 was reported to be involved in defense against the fungal pathogens *Botrytis cinerea* and *Alternaria brassicicola* (Faulkner et al., 2013; Narusaka et al., 2013). This might occur through control of symplastic fluxes in response to COs (Faulkner et al., 2013).

In *M. truncatula*, the only member of the phylogenetic group LYMII, MtLYM2 (*Medtr4g094730*) was expressed in BY-2 cells and was reported to bind long-chain COs (Fliegmann et al., 2011) but its affinity and involvement in CO responses have not been characterized.

Although all orthologs of OsCEBIP studied so far seem to have similar CO binding properties and to be involved in basal resistance to pathogenic fungi, they appear to be involved in various mechanisms. These mechanisms have been characterized only in rice and Arabidopsis. Studies in additional plant species are required to determine whether OsCEBIP orthologs are involved in defense mechanisms similarly to those found in rice or those found in Arabidopsis. Moreover the role of the second member of the phylogenetic group LYMII in monocotyledons needs to be determined.

#### LysM-RLK LYRI (LYK Clade I in Zhang et al., 2009)

Although absent in *A. thaliana* and *B. rapa*, all the plant species analyzed here have at least two members in the phylogenetic group LYRI, which can be divided into two subgroups here called A and B, each of them containing in most cases one member (Figure 4, Table 2, and Supplementary Figure S2). Some legumes have the particularity to possess two genes in the subgroup A (Gough and Jacquet, 2013). Members of the phylogenetic group LYRIA and especially the legume *MtNFP* (*Medtr5g019040*)/*LjNFR5* (*Lj2g3v1828350*) are also among the best-characterized LysM-RLK/Ps. They control Nod-factor perception and establishment of root endosymbioses.

**Table 2:** LysM-RLKs belonging to the phylogenetic groups LYRI, LYRII, LYRIII or LYRIV found in the 8 species analyzed.

		*M. truncatula*	*L. japonicus*	*P. persica*	*A. thaliana*	*B. rapa*	*S. lycopersicum*	*B. distachyon*	*O. sativa*
LYRI	A	Medtr5g019040	Lj2g3v1828350	Prupe.7G147300			Solyc02g065520	Bradi1g69290	Os03G13080
		MtNFP	LjNFR5	PpLYR1			SlLYK10	Bd LYR1	OsNFR5
		Medtr8g078300	Lj4g3v0912440						
		MtLYR1	LjLYS11						
	B	Medtr5G042440	^∗^	Prupe.1G027000			Solyc09g083210	Bradi3g51790	Os02G45750
		MtLYR8		PpLYR2			SlLYK9	Bd LYR2	
LYRII	A	Medtr7g029650	Lj1g3v3834250	Prupe.3G303700	At3g01840	Brara.E03634	Solyc02g094010		
		MtLYR10	LjLYS16	PpLYR6	AtLYK2		SlLYK2		
	B	Medtr4g058570	Lj3g3v3082380	Prupe.6G104800			Solyc11g069630		
		MtLYR9	LjLYS15	PpLYR7			SlLYK15		
LYRIII	A	Medtr5g019050	Lj2g3v1828320	Prupe.7G147600	At2g23770	Brara.D01416	Solyc02g089900	Bradi4g16350	Os11G35330
		MtLYR3	LjLYS12	PpLYR3	AtLYK4		SlLYK4	Bd LYR4	OsLYK6
	B	Medtr1g021845	Lj0g3v0145339	Prupe.7G147500			Solyc02g089920		
		MtLYR2		PpLYR4			SlLYK7		
							Solyc12g089020		
							SlLYK6		
	C	Medtr5g085790	Lj2g3v2899910		At2g33580	Brara.D02042		Bradi3g06770	Os06G41960
		MtLYR4	LjLYS13		AtLYK5			Bd LYR3	OsLYK3
		Medtr3g080170	Lj2g3v2899900						Os06G41980
		MtLYR7	LjLYS14						OsLYK2
									Os02G09960
									OsLYK4
LYRIV		Medtr7g079350	^∗^						
		MtLYR5							
		Medtr7g079320	Lj1g3v2808030	Prupe.6G357200					
		MtLYR6	LjLYS20	PpLYR5					

*^∗^Evidences for their existence (see Supplementary Table S1).*

##### Subgroup LYRIA

*MtNFP* and *LjNFR5* are required for the RNS in *M. truncatula* (Arrighi et al., 2006) and *L. japonicus* (Madsen et al., 2003), respectively. Almost no LCO response is detected in plants mutated in *MtNFP* or *LjNFR5*. Knockdown of *MtNFP* together with expression data suggest that it is involved in perception of Rhizobia all along the colonization process, including within nodules (Arrighi et al., 2006). Surprisingly, plants mutated in *MtNFP* have also been shown to be more sensitive to the pathogenic oomycetes *Aphanomyces euteiches* (Rey et al., 2013) and *Phytophthora palmivora* (Rey et al., 2015) and the fungal pathogens *Colletotrichum trifolii* (Rey et al., 2013) and *Verticillium albo-atrum* (Ben et al., 2013). Affinity for LCOs has been reported for LjNFR5 (Broghammer et al., 2012). LjNFR5 was expressed in a heterologous plant system (leaves of *Nicotiana benthamiana*), solubilized and purified to determine its affinity for an LCO with a structure close to the main Nod-factor produced by *Mesorhizobium loti* (the symbiotic partner of *L. japonicus* in RNS). High affinity was measured by SPR and MST, with a *Kd* of 4 and 10 nM, respectively. For SPR, an LCO derivative was immobilized on a chip and affinity determined using a range of LjNFR5 concentrations. For MST, affinity was determined using a fluorescent LCO derivative and a range of protein concentrations.

Whereas *MtNFP* and *LjNFR5* are not essential for establishment of AMS in legumes (Ben Amor et al., 2003; Madsen et al., 2003), the tomato ortholog, *SlLYK10* (*Solyc02g065520*) plays a role in AMS establishment (Buendia et al., 2016). Plants with decreased *SlLYK10* expression by VIGS showed a delay and less efficient colonization by the AMF *Rhizophagus irregularis*. In contrast, a rice knock-out mutant in the orthologous gene, *OsNFR5* (*Os03G13080*), was normally colonized by *R. irregularis* compared to WT plants although expression of AMS plant marker genes were reduced in the *Osnfr5* mutant compared to the WT, indicating a possible but weak role of *OsNFR5* in AMS establishment. Moreover, a chimera consisting of the sequences encoding LjNFR5 ECR and OsNFR5 ICR was able to complement the absence of nodulation in a *Ljnfr5* mutant, indicating that the function of the ICR is conserved between LjNFR5 and OsNFR5 (Miyata et al., 2016). Interestingly, in *Parasponia andersonii*, which belongs to a unique group of non-legume species able to form both the RNS with Rhizobia and the AMS, two members, resulting from a tandem duplication, are found in the phylogenetic group LYRIA (van Velzen et al., 2018). *PaNFP2* is closest to orthologs in legumes while *PaNFP1* is closest to orthologs in non-nodulating species. In non-nodulating species closely related to *P. andersonii* such as Trema species and in *P. persica*, the *PaNFP2* orthologs are truncated likely leading to non-functional proteins (van Velzen et al., 2018; Supplementary Table S1). *P. andersonii* plants containing a RNAi construct that might target the two paralogs, were affected in establishment of both RNS and AMS (Op den Camp et al., 2011). This suggests that an ancestral gene involved in LCO perception and AMS establishment was duplicated before the apparition of nodulation. One copy was then recruited during evolution for LCO perception in RNS establishment at least in legumes and *P. andersonii*. In some legumes, a second member of the phylogenetic group LYRIA is also found. *MtLYR1* (*Medtr8g078300*) in *M. truncatula* and *LjLYS11* (*Lj4g3v0912440*) in *L. japonicus* are the paralogs of *MtNFP* and *LjNFR5*, respectively. *MtLYR1* transcripts are detected in roots but not in nodules (Arrighi et al., 2006). During the AMS, *MtLYR1* transcripts increased in roots and more particularly cortical cells colonized by AMF (Gomez and Harrison, 2009). In *L. japonicus*, *LjLYS11* expression was not detected in roots and nodules but in cortical cells colonized by AMF. The expression patterns of *MtLYR1* and *LjLYS11* suggest a role in the AMS, possibly redundant with *MtNFP* and *LjNFR5*. However, *Ljlys11* single mutants and *Ljlys11-Ljnfr5* double mutants are colonized by AMF similarly to WT plants (Rasmussen et al., 2016).

In RNS, there is strong host specificity that is known to depend at least in part on LCO structure. Indeed, Rhizobia strains usually produce major LCO structures with particular decorations. These decorations distinguish them from each other. Members of the phylogenetic group LYRIA from legumes are thus expected to have selectivity for LCO structure. This hypothesis is supported by genetic studies consisting in heterologous expression of orthologous genes from plant species interacting with Rhizobia producing different LCO structures (Radutoiu et al., 2007; Bensmihen et al., 2011). In contrast, there is no strict host specificity for AMS, suggesting that legume members of the phylogenetic group LYRIA might have acquired the ability to discriminate LCO decorations while ancestor proteins involved in the AMS did not display this property. However, whether LjNFR5 that binds Nod-factor has selectivity for LCO structure has not been demonstrated yet.

Altogether, current data indicate that genes belonging to the phylogenetic group LYRIA are involved in root endosymbioses. This is coherent with their absence in Brassicaceae that do not establish RNS nor AMS. However, only partial or no deficiency of the AMS establishment was observed in plants with knock-down or knock-out for genes from the subgroup LYRIA in tomato, *P. andersonii*, rice and *L. japonicus* (Op den Camp et al., 2011; Buendia et al., 2016; Miyata et al., 2016; Rasmussen et al., 2016) suggesting a redundancy for activation of the CSSP (which is required for AMS establishment) possibly through perception of signals other than LCOs.

##### Subgroup LYRIB

All analyzed plant species have one gene in the subgroup LYRIB except in the Brassicaceae. In contrast to most LYRs, members of the phylogenetic group LYRIB have a unique intron. We found that in *L. japonicus*, the exons are split in two different loci: *Lj0g3v0102179* corresponds to exon 1 and *Lj0g3v0124999* corresponds to exon 2. For this reason the lotus gene was not included in the phylogenetic analysis. Although to date, no biological role and has been reported for members of this phylogenetic group, phylogenetic proximity to subgroup LYRIA and absence of member in *A. thaliana* and *B. rapa* make the members of the phylogenetic group LYRIB good candidates to play a role in AMS establishment in higher plants.

#### LysM-RLK LYRII (LYK Clade IV in Zhang et al., 2009)

This phylogenetic group was found only in dicotyledons and is divided into two subgroups here called A and B, each containing one member in the species analyzed except the group LYRIIB in which there are no members in the Brassicaceae (Figure 4, Table 2, and Supplementary Figure S2). In contrast with the other phylogenetic groups, the number and even the position of introns vary between orthologs.

#### LysM-RLK LYRIII (LYK Clades II and III in Zhang et al., 2009)

In the phylogenetic group LYRIII, several gene duplications occurred. On the basis of the phylogenetic analysis, we divided this phylogenetic group into three subgroups (Figure 4, Table 2 and Supplementary Figure S2). However, these three subgroups were only detectable when the phylogeny was performed without using the Gblock tool (an algorithm for curing the alignment and that restricts the phylogeny analysis to conserved regions). In fact, when Gblock was integrated in the analysis, the phylogenetic group LYRIII was divided between monocotyledonous and the dicotyledonous group members (Supplementary Figure S4). It is important to note that Gblock had no effect on the organization of the other phylogenetic groups (data not shown). This suggests that phylogeny of subgroups in the phylogenetic group LYRIII is not robust.

##### Subgroup LYRIIIA

All the species analyzed here possess members of the phylogenetic subgroup LYRIIIA. Members of this group *AtLYK4* (*At2g23770*) and *LjLYS12* (*Lj2g3v1828320*) were found to play a role in defense against pathogens. However, the biochemical characterization of MtLYR3 (Medtr5g019050) and LjLYS12 showed that these proteins are LCO binding proteins making difficult to understand the mechanisms in which they are involved.

In *A. thaliana*, responses to long-chain COs (CO6 and CO8) were decreased in *Atlyk4* knock-out mutants but not totally abolished, suggesting that AtLYK4 plays a role in long-chain CO perception (Wan et al., 2012). *AtLYK4* was also shown to play a positive role in defense against the fungal pathogen *Alternaria brassicicola* and the bacterial pathogen *P. syringae* (Wan et al., 2012). AtLYK4 was pulled down from *A. thaliana* solubilized membrane fractions using chitin beads and detected by mass spectrometry (Petutschnig et al., 2010; Wan et al., 2012). Affinity and selectivity of AtLYK4 for COs is unknown. Because the responses to long-chain COs were not abolished in *Atlyk4* mutants, the authors suggested that an additional protein plays a redundant role in long-chain CO perception. This protein might be AtLYK5 described below. However, the implication of *AtLYK4* in resistance to a bacterial pathogen questions the possible function of AtLYK4 as a CO binding protein and suggests a more general role in MAMP perception.

In *L. japonicus*, *LjLYS12* (*Lj2g3v1828320*) expression is induced during infection by the oomycete *Phytophthora palmivora* and plant knock-outs for *LjLYS12* are more susceptible to *P. palmivora* while no difference in RNS and AMS was detected compared to WT plants (Fuechtbauer et al., 2018). In *M. truncatula*, the biological role of *MtLYR3* (Medtr5g019050) still remains unknown, however, Fliegmann et al. (2013) demonstrated that MtLYR3 has a high affinity for LCOs (*Kd* around 25 nM) reminiscent of a binding site characterized in a *Medicago varia* cell culture (Gressent et al., 1999). The protein was expressed in *N. benthamiana* leaves and LCO binding assays were performed on membrane fractions by competition between radiolabeled LCOs at a fixed concentration and ranges of concentrations of various unlabeled LCOs or COs. The MtLYR3 LCO binding site is specific for LCOs versus COs, however, it does not discriminate LCO decorations on the GlcNAc backbone. MtLYR3 orthologs in other legumes including LjLYS12, display similar affinities for LCOs as MtLYR3 except those of two *Lupinus* species incapable of forming the AMS, that do not bind LCOs (Malkov et al., 2016). This suggests that these proteins could play a role in the AMS at least in legume plants.

Interestingly, genes from the phylogenetic groups LYRIA and LYRIIIA are located at the same locus, as neighboring genes in opposite orientations in most dicotyledons (Figure 5). LCO binding properties of the proteins from these two phylogenetic subgroups are likely the consequence of a tandem duplication of an ancestral gene encoding a LCO binding protein.

In conclusion, members of the phylogenetic subgroup LYRIIIA appear to be involved in defense mechanisms while at least in legumes they can bind LCOs with high affinity. Additional studies are required to understand whether these genes could be involved in crosstalk between LCO perception and defense regulation.

##### Subgroup LYRIIIB

There is at least one member of the phylogenetic subgroup LYRIIIB in the genome of all the analyzed dicotyledons, except in the Brassicaceae. These genes are located next to the genes from the phylogenetic groups LYRIIIA in most dicotyledons except in legumes (Figure 5).

##### Subgroup LYRIIIC

Members of the phylogenetic subgroup LYRIIIC were not found in all the species analyzed here (they are absent in peach and tomato). *AtLYK5* (*At2g33580*) and *MtLYR4* (*Medtr5g085790*) are the best-characterized members of this phylogenetic group. They are involved in the perception of long-chain COs and resistance against fungal pathogens. An *Atlyk5* knock-out mutant is strongly, although not fully, inhibited in responses to long-chain COs (CO6 to CO8) elicitation and is more susceptible to the fungus *A. brassicicola* (Cao et al., 2014). In contrast, the double mutant *Atlyk4*/*Atlyk5* has completely abolished responses to long-chain COs but has a susceptibility to *A. brassicicola* similar to the *Atlyk5* knock-out mutant (Cao et al., 2014). Affinity of AtLYK5 for COs was measured by ITC on AtLYK5 ECR produced in *E. coli*. An affinity of 1.72 μM was found for CO8 while no binding to CO4 was detected. Using mutated versions of AtLYK5, it was shown that its CO binding activity is essential for its biological role. Key residues in the AtLYK5 CO binding site were identified by comparison with Ecp6, a fungal secreted protein containing 3 LysMs, which binds CO8 with high affinity (see below). Mutation in AtLYK5 Y128 and S206 led to inability of the tagged protein produced *in planta* to bind chitin beads and to the corresponding coding sequence to restore the ROS production in response to chitin when used to complement *Atlyk5* plants (Cao et al., 2014).

Two members of the phylogenetic subgroup LYRIIIC are found in legumes. In *M. truncatula* the *MtLYR4* (*Medtr5g085790*) and *MtLYR7* (*Medtr3g080170*) are found on different chromosomes while in *L. japonicus LjLYS13* (*Lj2g3v2899910*) and *LjLYS14* (*Lj2g3v2899900*) are closely related genes, suggesting a more recent duplication event in *L. japonicus*. *Mtlyr4* mutants showed increased susceptibility to the fungal pathogen *B. cinerea* (Bozsoki et al., 2017) and a loss of ROS production induced by CO4 or CO8. Furthermore, *Mtlyr4* mutants showed a decreased MAPK 3/6 phosphorylation (a hallmark of MTI signaling) compared to WT when treated with 1 μM CO8. *LjLYS13* is expressed particularly in roots and up-regulated by CO8 treatment (Lohmann et al., 2010), suggesting a role in CO perception. *LjLYS14* (*Lj2g3v2899900*) is expressed more ubiquitously and is also slightly induced by CO8. *LjLYS13* and *LjLYS14* expression is up-regulated in roots in presence of Rhizobia but not detected in nodules (Lohmann et al., 2010). In contrast to *Mtlyr4* mutants, ROS production was similarly induced by CO4 or CO8 in *Ljlys13 or Ljlys14* mutants as in WT plants (Bozsoki et al., 2017). This could be due to redundant functions of LjLYS13 and LjLYS14. Since it unlikely to obtain a double mutant by crossing single mutants because of the close proximity of the genes, it would be very informative to obtain the double mutant, for example using the CRISPR-Cas9 technology. The difference in the responses of *Mtlyr4*, *LjLys13*, and *Ljlys14* to CO4 or CO8, reinforces the hypothesis that duplication events in the phylogenetic subgroup LYRIIIC were independent in *M. truncatula* and *L. japonicus*. In addition, MtLYR4 phosphorylation status was found to be affected by LCO treatment in a *MtNFP*-independent manner (Rose et al., 2012). Although the effect of LCOs on MtLYR4 phosphorylation is CSSP-independent, the MtLYR4 phosphorylation status itself appears to be controlled by the CSSP (Rose et al., 2012). However, *Mtlyr4* mutants (as *Ljlys13* and *Ljlys14* mutants) are not affected in the RNS (Bozsoki et al., 2017).

#### LysM-RLK LYRIV (Not Named in Zhang et al., 2009)

Among the species analyzed, the phylogenetic group LYRIV contains members only in legume species and in peach (Figure 4, Table 2, and Supplementary Figure S2), suggesting that this group emerged in a common ancestor to these closely related plant species. Two members are found in *M. truncatula* and were reported in *L. japonicus* (Lohmann et al., 2010). Their kinase domains are very different from the other LYRs and closely related to RLKs from the wall associated kinase (WAK) subfamily (Arrighi et al., 2006).

#### LysM-RLK LYKI (LYK Clade VI in Zhang et al., 2009)

In the phylogenetic group LYKI the number of genes is highly variable between species (Figure 6, Table 3, and Supplementary Figure S3). Legumes display the highest number and diversity of members in this phylogenetic group (9 in *M. truncatula* and 5 in *L. japonicus*) whereas we only found 1 member in Brassicaceae and in *B. distachyon*. Members of this phylogenetic group are involved in the perception of the various GlcNAc-containing ligands (at least CO and PGN) and have dual roles in endosymbiosis and defense. They might be co-receptors rather than ligand-binding proteins.

**Table 3:** LysM-RLKs belonging to the phylogenetic groups LYKI, LYKII or LYKIII found in the 8 species analyzed.

	*M. truncatula*	*L. japonicus*	*P. persica*	*A. thaliana*	*B. rapa*	*S. lycopersicum*	*B. distachyon*	*O. sativa*
LYKI	Medtr5g086540	Lj2g3v2904640						
	LYK1	LjLYS2						
	Medtr5g086120							
	LYK4							
	Medtr5g086090							
	LYK5							
	Medtr5g086040	Lj2g3v2904610						
	LYK6	LjLYS1						
	Medtr5g086030							
	LYK7							
	Medtr5g086310 Medtr5g086330							
	LYK2							
	Medtr5g086130	Lj2g3v2904690				Solyc01g098410		
	LYK3	LjNFR1				SlLYK13		
	Medtr3g080050	Lj6g3v1055580	Prupe.3G213100			Solyc07g049180		
	LYK9	LjLYS6	PpLYK2			SlLYK1/SlBti9		
	Medtr2g024290	Lj6g3v1812110	Prupe.4G016100			Solyc02g081050	Bradi3g41590	Os08g42580
	LYK8	LjLYS7	PpLYK1			SlLYK12	BdLYK1	OsCERK1
						Solyc02g081040		Os09g33630
						SlLYK11		
				At3g21630	Brara.E02055			
				AtLYK1/AtCERK1				
LYKII	Medtr5g033490	Lj2g3v1415410	Prupe.5G168000			Solyc06g069610	Bradi2g40627	Os01G36550
	LYK10	LjLYS3/EPR3	PpLYK3			SlLYK14	BdLYK2	OsLYK1
			Prupe.1G247900					
			PpLYK4					
LYKIII	Medtr8g014500	Lj3g3v2318170	Prupe.3G058700	At1g51940	Brara.H00193	Solyc03g121050	Bradi2g49400	Os01G53840
	LYK11	LjLYS5	PpLYK5	AtLYK3		SlLYK3	BdLYK3	
		Lj3g3v0290100			Brara.F00223			
		LjLYS4						

This phylogenetic group contains *AtLYK1*/*AtCERK1* (*At3g21630*) that has been widely studied and first shown to be required for chitin responses. CO8-induced responses such as ROS production and MAP kinase phosphorylation are completely impaired in *Atcerk1* knock-out mutants (Miya et al., 2007; Wan et al., 2008).

Contrasted results have been obtained concerning the affinity of AtCERK1 for chitin and COs. High affinity for chitin (*Kd* of 2 nM) has been reported (Iizasa et al., 2009) for the full length protein fused to GFP, produced in yeast, solubilized and purified to measure binding on chitin beads using a range of protein concentration. However, in the same study competition assays on the chitin beads using CO5, CO6, or CO8 led to half-maximal inhibitory concentration (IC50) of about 100 μM. Similarly, other studies showed a low affinity binding (*Kd* of 44 and 455 μM for CO8) using ITC with purified AtCERK1 ECR produced in insect cells and in *E. coli*, respectively (Liu T. et al., 2012; Cao et al., 2014). The huge differences between affinity reported for chitin and COs could be due to the methods used for affinity determination (quantification of AtCERK1:GFP fluorescence bound to chitin beads and ITC) or to differences in affinity for various degrees of GlcNAc polymerization (chitin vs. CO8).

Although AtCERK1 has been mainly studied for its role in chitin perception, it was shown that a *Atcerk1* knock-out line is more sensitive to the pathogenic bacterium *P. syringae* (Wan et al., 2012) suggesting that AtCERK1 is also involved in perception of bacterial MAMPs. Indeed, AtCERK1 has been shown to be involved in PGN perception (Willmann et al., 2011) although it does not appear to directly bind PGN (Petutschnig et al., 2010; Willmann et al., 2011).

Similarly, tomato lines with reduced expression of *SlLYK1*/*Bti9* (*Solyc07g049180*) and *SlLYK3* (*Solyc01g098410*) were more susceptible to *P. syringae* (Zeng et al., 2012).

Finally, *AtCERK1* was recently shown to be involved in perception of β-1,3 glucan hexasaccharides, which is not a GlcNAc-containing molecules (Mélida et al., 2018). Consistent with this observation, the *Atcerk1* mutant was more susceptible to the oomycete *Hyaloperonospora arabidopsidis*, the cell wall of which is devoid of chitin (Mélida et al., 2018).

OsCERK1 (Os08g42580) is also involved in chitin and PGN signaling (Shimizu et al., 2010; Ao et al., 2014). Similarly to what has been observed for the *Atcerk1* mutant, *Oscerk1* mutants display strongly reduced responses to chitin, soluble CO7-8 or PGN treatment (ROS production, apoplastic alkalinization, genes regulation and callose deposition). As expected for a chitin-perception defective mutant, *Oscerk1* mutant is more susceptible to the fungal pathogen *M. oryzae* (Kouzai et al., 2014a). However, unlike AtCERK1, OsCERK1 did not show any binding to insoluble colloidal chitin (Shinya et al., 2012). This difference of the biochemical properties between AtCERK1 and OsCERK1 was supported by the fact that OsCERK1 could not complement *Atcerk1* for CO8-induced ROS responses (Shinya et al., 2012). Chimera consisting in AtCERK1 ECR and OsCERK1 ICR was able to partially rescue ROS production, suggesting that AtCERK1 binding properties are necessary for ROS responses in Arabidopsis.

Interestingly, an *Oscerk1* knock-out line displayed a mycorrhizal phenotype (Miyata et al., 2014) with no root colonization at 15 days post inoculation (dpi), demonstrating a role of *OsCERK1* in early fungal colonization. Some penetration sites and arbuscules were observed at 30 dpi. Similarly, rice plants with decreased level of *OsCERK1* showed almost no AMF penetration at 6 weeks post inoculation (wpi; Zhang et al., 2015). This suggests that OsCERK1 is involved in perception of signals produced by AMF. Indeed, *OsCERK1* is required for CO4 and CO5 perception as these molecules were unable to induce Ca^2+^ responses in *Oscerk1* whereas *Oscebip* and *Osnfr5* still display calcium spiking (Carotenuto et al., 2017).

Finally, *OsCERK1*, but not *AtCERK1*, was recently shown to be involved in perception of LPS, which is also not a GlcNAc-containing molecules (Desaki et al., 2018).

There is another member of the phylogenetic group LYKI in rice, *OsRLK10* (*Os09g33630*). It would be interesting to determine whether *OsRLK10* is functionally redundant with *OsCERK1* for one or both of the *OsCERK1* functions.

Recently, Bozsoki et al. (2017) showed that members of the phylogenetic group LYKI in legumes, *LjLYS6* (*Lj6g3v1055580*) and *MtLYK9* (*Medtr3g080050*) are involved in defense. *Ljlys6* and *Mtlyr9* knock-out mutants are more susceptible than WT plants to the fungal pathogen *B. cinerea.* Moreover, responses to a range of COs (from CO4 to CO8) such as ROS production or MAPK3/6 phosphorylation were decreased compared to WT. To determine LjLYS6 affinity for COs, LjLYS6 ECR was produced in insect cells. After purification, it was deglycosylated and labeled with a fluorophore. Affinity for CO5 to CO8 was measured by MST using a range of CO concentrations. LjLYS6 was found to have a higher affinity for COs with long chains than with short chains, with a *Kd* of 38 μM for CO8, 227 μM for CO5 and no detectable binding of CO4. The affinity for CO8 is comparable to that found for AtCERK1 ECR produced in insect cells and measured by ITC (Liu T. et al., 2012). The crystal structure of LjLYS6 ECR (Bozsoki et al., 2017) was found to be similar to that of AtCERK1 ECR (Liu T. et al., 2012) although the authors could not observe LjLYS6 ECR bound to COs.

Other members of the phylogenetic group LYKI in legumes, *LjNFR1* (*Lj2g3v2904690*) and *MtLYK3* (*Medtr5g086130*) are involved in LCO (Nod-factor) perception in the RNS. The genes originate from duplication events specific to legumes (De Mita et al., 2014). *Ljnfr1* mutants were impaired in nodulation and in the earliest responses to LCOs (Radutoiu et al., 2003). Apoplast alkalinization, that occurs immediately after LCO application and later responses such as root hair deformation (few hours after LCO application), were not observed in *Ljnfr1* mutant lines. Whether *LjNFR1* also plays a role in the AMS is a matter of debate. It has been shown that *Ljnfr1* mutant lines display a lower colonization ratio compared to the WT 5 wpi (Zhang et al., 2015). Moreover, AMS marker genes and Myc-LCO-induced calcium spiking were reduced in an *Ljnfr1* mutant line compared to the WT (Zhang et al., 2015). In contrast, no difference in colonization ratio or fungal structure morphology was observed between a triple *Ljnfr1-Ljnfr5-LjLys11* mutant line and the WT (Rasmussen et al., 2016). LjNFR1 LCO binding was analyzed with the same strategy as for LjNFR5 (phylogenetic group LYRIA). High affinity for LCO structure derivatives from *M. loti* main LCOs was found with a *Kd* of 4.9 nM using SPR and with a *Kd* of 0.61 nM using MST (Broghammer et al., 2012).

**FIGURE 7:**
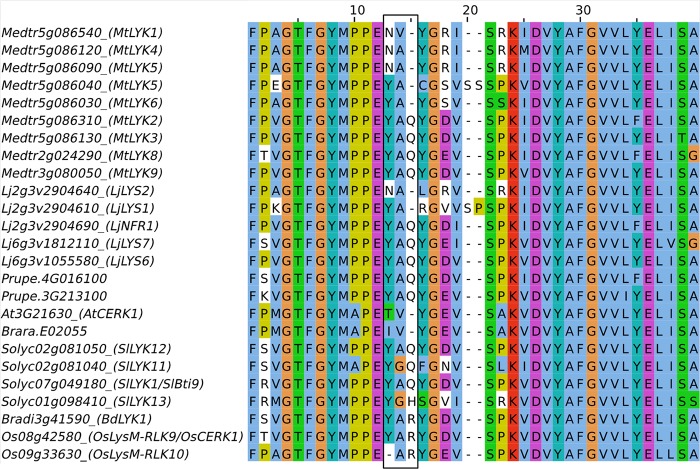
Partial amino acid sequence alignment of members of the phylogenetic group LYKI. The YAQ/R motif present in the kinase domain is boxed in black.

Structural differences were found between the LysM-RLKs of the phylogenetic group LYKI from species that establish endosymbiosis and those of the Brassicaceae which do establish endosymbioses. In all species except in the Brassicaceae, there is at least one member of the phylogenetic group LYKI that contains a specific motif in the kinase domain, YAQ in dicotyledons and YAR in monocotyledons, while AtCERK1 and Brara.E02055 have, respectively, one member that contains the residues TV or IV instead at this position (Figure 7). The YAQ/R motif has been demonstrated to be important for nodulation. Expression of a chimera containing the LjNFR1 ECR and the AtCERK1 ICR in a *Ljnfr1* mutant was unable to restore nodulation (Nakagawa et al., 2011) in contrast to a LjNFR1-OsCERK1 chimera (Miyata et al., 2014). Replacement in AtCERK1 of the residues TV by YAQ led the chimera LjNFR1-AtCERK1^TV -Y AQ^ to restore nodulation in *Ljnfr1*. This suggests that the YAQ/R motif is associated with a symbiotic function either in the RNS as in LjNFR1 or in the AMS as in OsCERK1. However, in the dicotyledonous species analyzed that establish AMS, at least two paralogs bear the YAQ/R motifs and might have redundant roles (Figure 7). Since RNS is completely abolished in the *Ljnfr1* mutant, it is unlikely that another LysM-RLK has a redundant function in RNS. While it bears the YAQ/R motif, LjLYS6 was not found to be involved in the RNS nor in the AMS (Bozsoki et al., 2017). It could be hypothesized that LjLYS6 and LjLYS7 which both bear the YAQ/R motif have a redundant role in AMS establishment.

A knock-down of the *LjLYS6* ortholog in pea, *PsLYK9*, conferred sensitivity to the fungal pathogen *Fusarium culmorum*. In addition, roots were also affected in transcriptomic responses to CO5, including the expression of genes that are upregulated in WT roots colonized by AMF in pea suggesting that *PsLYK9* is involved in symbiosis signaling (Leppyanen et al., 2017).

The *LjNFR1* ortholog in *M. truncatula*, *MtLYK3*, has been demonstrated to be involved in nodulation. *Mtlyk3* knock-down (Limpens et al., 2003) or missense (Wais et al., 2000; Catoira et al., 2001; Smit et al., 2007) lines were impaired in nodule formation and in Rhizobial colonization of *M. truncatula* roots but not in LCO responses such as apoplast alkalinization or calcium spiking. This led to the hypothesis that the genetic control of Rhizobial colonization is different between *M. truncatula* and *L. japonicus* and that *MtLYK3* is involved LCO perception during Rhizobial colonization but not in LCO perception preceding colonization. This was consistent with the hypothesis of there are two receptors controlling responses to Nod-factors and rhizobial colonization in *M. truncatula* (Ardourel et al., 1994). However, a tandem duplication of *MtLYK3* has occurred in *M. truncatula.* Two genes *MtLYK3* and *MtLYK2* (Medtr5g086310/Medtr5g086330) are *LjNFR1* orthologs (Figure 6; De Mita et al., 2014). *MtLYK2* might be redundant with *MtLYK3.* Actually, MtLYK2 contains as MtLYK3, the YAQ/R motif in its kinase domain. Although *MtLYK2* is less expressed than *MtLYK3* in roots (Limpens et al., 2003), it is possible that the *MtLYK2* expression level in *Mtlyk3* mutant lines is enough to ensure LCO responses preceding colonization but not Rhizobial colonization. This would explain the phenotypic difference between *Mtlyk3* and *Ljnfr1.* Consistently, complementation experiments have shown that a chimeric protein containing LjNFR1 ECR and MtLYK3 ICR can restore the absence of nodulation in a *Ljnfr1* mutant (Nakagawa et al., 2011) suggesting a conservation of the function of these legume LysM-RLKs.

LjNFR1 and MtLYK3 are located in a cluster that contains 3 LYK genes in *L. japonicus* and 7 in *M. truncatula*. The number of LYK genes in this cluster is highly variable between legume species and could be partly responsible for host specificity through adaption to variation in the Nod-factor structure secreted by the Rhizobial symbionts as suggested by work in pea (Sulima et al., 2017).

Taken together, these data suggest evolution of members of the phylogenetic group LYKI for which the ancestral protein might have had a dual role in defense and AMS. Such a dual function is still found in rice. In this scenario, proteins have been subfunctionalized for a role in defense in Brassicaceae. In the other dicotyledonous species, the genes experienced several duplication events, which likely led to redundancy for a role in AMS and neofunctionalization for a role in RNS in legumes. Finally, because individual member of the phylogenetic group are involved in the perception of various molecules (at least PGN, long-chain COs and in β-1,3-glucan for AtCERK1, PGN, all COs and LPS for OsCERK1) leading to different biological responses and because they bear an active kinase in contrast to the LYRs, it is likely that these proteins are essential for signaling rather than for the specificity of ligand perception. This point will be discussed in the “Hetero-oligomeric complexes” section.

#### LysM-RLK LYKII (Not Named in Zhang et al., 2009)

In the Phylogenetic group LYKII, we found one ortholog in each species analyzed, except in the Brassicaceae (Figure 6, Table 3, and Supplementary Figure S3). In the peach genome, gene duplication occurred and two copies are present. The only characterized member of the phylogenetic group LYKII is the *L. japonicus* member *LjEPR3*/*LjLYS3* (*Lj2g3v1415410*) which has been shown to be implicated in the recognition of bacterial EPS and colonization by Rhizobia (Kawaharada et al., 2015, 2017). Knock-out or missense *Ljepr3* mutants developed more nodules (although the number of nodules was extremely low) in the presence of a Rhizobial *M. loti* strain that produces an abnormal EPS structure (*exoU*) and which is almost unable to colonize WT *L. japonicus* (Kawaharada et al., 2015). In contrast to the *M. loti exoU* strain, a *M. loti* strain unable to produce EPS (*exoB*) was able to colonize WT *L. japonicus*, despite plants showed abnormal ITs (the cell invaginated structure that allow Rhizobia colonization in nodules) and intercellular Rhizobial colonization in nodules. Although to a lower extent, this phenotype was also observed in the *Ljepr3* mutants inoculated with WT *M. loti*. The quantitative phenotypic differences between *Ljepr3* and WT plants suggest that EPR3 is, however, not the only actor in EPS perception (Kawaharada et al., 2017). *LjEPR3* expression is induced by Nod-factors and by Rhizobia. In the presence of Rhizobia, its expression pattern in roots corresponds to the zone susceptible to Rhizobial colonization, around the ITs and in the nodule primordia. This suggests that *LjEPR3* is required all along the infection process (Kawaharada et al., 2017).

The orthologous gene in *M. truncatula*, *MtLYK10* (*Medtr5g033490*) is also induced by Nod-factors and Rhizobia, and by Myc-factors and during the AMS (Mt gene atlas, Mtr.25148.1.S1_at; Camps et al., 2015). Similarly, orthologs in monocotyledons (*Os01g36550* and *Bradi2g40627*) are induced during the AMS (Güimil et al., 2005, gene annotated *OsAM191*; and personal communication). The absence of any member of the phylogenetic group LYKII in Brassicaceae, together with the role of *LjEPR3* in Rhizobial colonization and the induction of the expression of various orthologs in the presence of root symbionts, suggest that the members of this phylogenetic group play a role in root endosymbioses. However, it has recently been reported that there is no ortholog of LjEPR3 in *P. andersonii* (van Velzen et al., 2018). LjEPR3 ECR was expressed in insect cells by using a baculovirus system. Binding to EPS was measured by biolayer interferometry using purified LjEPR3 ECR (Kawaharada et al., 2015) and a *Kd* of 2.7 μM was found. Whether the recognition of EPS by LysM-RLKs is specific to *L. japonicus* or legumes remains unknown. It is therefore of interest to study a putative role in the AMS of non-legume members of the phylogenetic group LYKII and to determine their biochemical properties, especially their ability or not to bind bacterial EPS.

#### LysM-RLK LYKIII (LYK Clade V in Zhang et al., 2009)

In the phylogenetic group LYKIII, we identified at least one ortholog in each species, with duplications in *B. rapa* and in *L. japonicus* (Figure 6, Table 3, and Supplementary Figure S3). The only gene from this phylogenetic group that has been studied is *AtLYK3* (*At1g51940*) and it was shown to act as a negative regulator of plant immunity in *A. thaliana* (Paparella et al., 2014). A T-DNA insertional mutant line displayed reduced symptoms in the presence of the fungal pathogen *B. cinerea* or the bacterial pathogen *Pectobacterium carotovorum* when compared to WT plants. In addition, basal expression in absence of pathogen of defense-related genes such as *PAD3*, a gene involved in phytoalexin biosynthesis, was higher in *Atlyk3* mutants than in WT plants. *AtLYK3* was also shown to be required for LCO perception in *A. thaliana* (Liang et al., 2013). The authors showed that treatment with LCO (at 100 nM) or CO4 (at 10 μM) partially inhibits (about 25%) *A. thaliana* WT responses to the MAMPs flg22 or CO8. LCO effects on the attenuation of flg22 responses seem to occur through degradation of AtFLS2. LCO effects on flg22-induced ROS production were not observed in an *Atlyk3* knock-out line and were stronger in an *AtLYK3* overexpressing line suggesting that AtLYK3 is involved in LCO perception. Note that the data also suggest that in this *Atlyk3* knock-out line, flg22-induced MAP kinase phosphorylation was reduced in absence of LCO.

## General Discussion on LysM-Rlk Roles and Functions

### LysM-RLKs Function as Hetero-Oligomeric Complexes

It is considered that RLKs function as hetero-oligomers composed of at least one protein that bind a signal molecule with high affinity through its ECR and one protein that transduce the signal through an active kinase domain in its ICR. The receptors for the MAMP peptides flg22 and elf18 occurs through high affinity binding to the LRR-RLKs AtFLS2 and AtEFR, respectively, and subsequent complex formation with the LRR-RLK AtBAK1 (Chinchilla et al., 2007; Schwessinger et al., 2011). AtFLS2 and AtEFR have non-RD kinases with weak activity (Schwessinger et al., 2011) compared to AtBAK1 which has a RD kinase and which is involved in multiple signaling pathways. AtFLS2 and AtEFR are then internalized following ligand perception in an AtBAK1-dependent manner (Mbengue et al., 2016).

**FIGURE 8:**
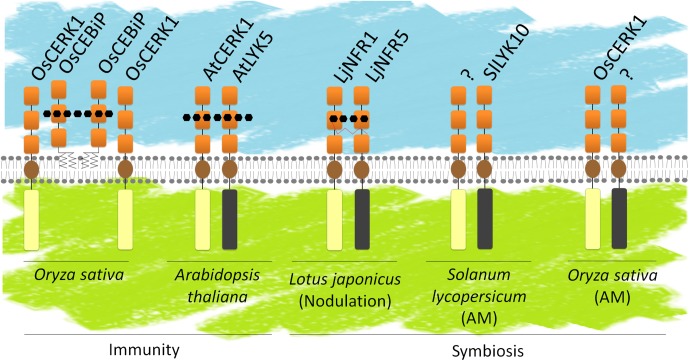
LysM-RLK and/or LysM-RLP heterodimers. Known or hypothetical heterodimers involved in defense or symbiosis. Schematic representation of the LysM-RLKs/Ps as in Figure 1. LysM-RLKs with beige ICRs are LYKs (with active kinase domains), LysM-RLKs with gray ICRs are LYRs (with inactive kinase domains). Several models have been proposed in the literature: OsCEBIP might from a dimer that binds CO8 and interacts with OsCERK1 for signaling; AtLYK5 and AtCERK1 might both bind CO8; LjNFR5 and LjNFR1 might both bind LCOs. SlLYK10 and OsCERK1 might interact with yet unidentified partners for perception of Myc-factors.

A model for ligand perception by LysM-RLK/Ps proposes hetero-oligomers composed of at least one LYR/LYM and one LYK (Figure 8). It can be hypothesized that LYR or LYM proteins, lacking active kinase domain, are the partners that bind signal molecules with high affinity through their ECRs. High affinity likely corresponds to *Kd* values in the range of nM as measured for several LYRs and LYMs. Ligand binding to a LYR/LYM would induce (i) interaction with a LYK, which possesses an active kinase, or (ii) a change of conformation of the pre-existing LYR/LYM and LYK complex, leading to activation of the kinase of the LYK partner and signal transduction. Fitting this model, the LYMs OsCEBIP, OsLYP4, OsLYP6, AtLYM1, AtLYM2, AtLYM3, and MtLYM2 were found to bind PGN and/or COs and the LYRs AtLYK5, MtLYR3, LjLYS12, and LjNFR5 were shown to bind COs or LCOs. Except for OsLYP4 and OsLYP6, these proteins showed selectivity for a single type of ligand. Moreover, when their affinity was measured, these proteins were found to have high affinity for ligands. In contrast, the LYKs AtCERK1 and OsCERK1 were found to be involved in perception of multiple signals and to have low or no affinity for GlcNAc-containing ligands. Many genetic analyses actually suggest that LYMs/LYRs and LYKs interact, since mutants show similar phenotypes in responses to molecules or microorganisms (Table 4). Supporting the requirement of heterodimeric receptors to bind a ligand and transduce the signal, changes in host range during RNS or ligand specific responses were obtained by heterologously expressing couples of LYR/LYM and LYK proteins. Co-expression of LjNFR5 (LYRIA) and LjNFR1 (LYKI) in *M. truncatula* or in *Lotus filicaulis* modified host range (Radutoiu et al., 2007), while single proteins did not. Co-expression of chimeric LjNFR5–AtCERK1 and LjNFR1–AtCERK1 in *A. thaliana* led to production of ROS and expression of chitin-induced genes in response to LCO (Wang et al., 2014). Similarly, co-expression of chimeric OsCEBiP–LjNFR5 and OsCERK1–LjNFR1 in *L. japonicus* led to induction of LCO responsive genes in response to chitin and CO8 (Wang et al., 2014). Finally, physical interactions between LYMs/LYRs and LYKs have been demonstrated *in planta*. In rice cells, OsCEBIP, OsLYP4, and OsLYP6 (LYMII) interact with OsCERK1 (LYKI) in the presence of chitin (Shimizu et al., 2010; Ao et al., 2014). OsLYP4 and OsLYP6 also interact with OsCERK1 in presence of PGN (Ao et al., 2014). In *A. thaliana*, AtLYK5 (LYRII) interacts with AtCERK1 (LYKI), (Cao et al., 2014) under CO elicitation (CO6, CO7 and CO8 but not CO5). In *M. truncatula* physical interaction between MtNFP (LYRIA) and MtLYK3 (LYKI) was shown in nodules (Moling et al., 2014). It is unknown whether this interaction requires a ligand, although there is evidence for Nod-factor production by rhizobia inside legume nodules. Physical interaction of LjNFR5 (LYRIA) with LjNFR1 (LYKI) was shown in the absence of Nod-factors when expressed in a heterologous system (Madsen et al., 2011). However, it has to be noted that this was in a context of high expression levels. Physical interaction was also shown for MtLYR3 (LYRIIIA) and MtLYK3 (LYKI) in a heterologous system and in the absence of ligand and this interaction was decreased in the presence of LCO (Fliegmann et al., 2016).

**Table 4:** Known or hypothetical LysM-RLK/P heterodimers involved in defense or symbiosis.

	Genetic interaction	Reference	Physical interaction	Reference
AtLYM3/AtCERK1	X	Willmann et al., 2011		
AtLYM1/AtCERK1	X	Willmann et al., 2011		
AtLYK4/AtCERK1	X	Wan et al., 2012; Cao et al., 2014		
AtLYK5/AtCERK1	X	Cao et al., 2014	X	Cao et al., 2014
OsLYP4/OsCERK1	X	Liu B. et al., 2012	X	Liu B. et al., 2012; Ao et al., 2014
OsLYP6/OsCERK1	X	Liu B. et al., 2012	X	Liu B. et al., 2012; Ao et al., 2014
OsCEBIP/OsCERK1	X	Ao et al., 2014; Kouzai et al., 2014a; Liu et al., 2016	X	Ao et al., 2014; Hayafune et al., 2014; Liu et al., 2016
MtNFP/MtLYK3	X	Limpens et al., 2003; Arrighi et al., 2006	X	Moling et al., 2014
LjNFR5/LJNFR1	X	Madsen et al., 2003; Radutoiu et al., 2003	X	Madsen et al., 2011
MtLYR3/MtLYK3			X	Fliegmann et al., 2016

After ligand perception, some LysM-RLKs might be internalized in endosomes for activation of signaling events taking place in endosomes and/or deactivation and degradation in the vacuole. This is the case for AtLYK5 internalization, which is induced by treatment with chitin. In contrast, AtCERK1 endocytosis does not seem to de induced by ligand perception. However, AtLYK5 phosphorylation and internalization depend on AtCERK1 kinase activity upon chitin treatment (Erwig et al., 2017).

Although most data on LysM-RLK/Ps fit with the model of a receptor complex consisting in a ligand binding protein and a protein involved in signaling, high affinity LCO binding of the kinase active LjNFR1 (LYKI) questions the model. Other high affinity ligand-binding proteins such as the LRR-RLK AtPERP1/2 which are receptors of endogenous peptides involved in wound signaling and innate immunity (Yamaguchi et al., 2006; Yamaguchi et al., 2010) and the LRR-RLK AtBRI1 which is the receptor of the Brassinosteroid hormone (He et al., 2000) have an active RD kinase. Recent data showing the involvement of OsCERK1 in perception of LPS and AtCERK1 in perception of β-1,3-glucan also open the possibility that LYKs participate to receptor complexes with other type of proteins than LysM-RLK/Ps. Since LysM-RLKs are not expected to bind with high affinity to LPS, β-1,3-glucans or EPS and no LYR or LYM was found up to now to be involved in perception of these molecules, it is likely that AtCERK1, OsCERK1, and LjEPR3 interact with members of other protein families to form high affinity receptors for these molecules.

### Limitations in Ligand Binding Assays

As reported above, the biochemical assays performed to characterize LysM-RLK/Ps have produced contrasting results. Differences in affinity for ligands of a LysM-RLK/P found between studies might be due to various reasons including the production system, the region of the protein used (e.g., full length versus ECR) and the binding assay. Because the *E. coli* system is not efficient for the formation of disulfide bridges that were shown to be essential for the function of several LysM-RLKs including MtNFP (Lefebvre et al., 2012) and LjEPR3/LjLYS3 (Kawaharada et al., 2015), the affinity measured with ECRs produced in *E. coli* might be underestimated. Indeed the AtCERK1 ECR produced in insect cells showed a *Kd* for CO8 of 44 μM by ITC (Liu T. et al., 2012) while AtCERK1 ECR produced in *E. coli* showed a *Kd* for CO8 of 455 μM (Cao et al., 2014) using the same binding assay. However, differences were also found using various expression systems allowing formation of disulfide bridges. Expression of OsCEBIP full length protein in tobacco BY-2 cells suggested a *Kd* for CO8 around 100 nM, while using the OsCEBIP ECR produced in insect cells, the *Kd* for CO8 was about 4 μM. Differences affinity for ligands have also been found for the fungal effector Ecp6, a protein containing 3 LysMs which binds CO8. Using a recombinant Ecp6 purified from *Pichia pastoris Kd* between 3.7 and 4.5 μM were found by ITC (de Jonge et al., 2010; Mentlak et al., 2012) while *Kds* of 1.3 nM or 380 nm were found by SPR using a CO8 immobilized or an effector-immobilized strategy, respectively. Using a recombinant Ecp6 purified from mammalian cells, a *Kd* of 280 pM was found using ITC (Sánchez-Vallet et al., 2013). One reason explaining the differences between the results obtained with recombinant Ecp6 from yeast or mammalian cells might be the presence of yeast-derived COs co-purified with the protein that biased the analysis (Sánchez-Vallet et al., 2013). It is thus essential to compare affinities of proteins or domains produced similarly and characterized using the same binding assay.

Although half maximal incorporation in saturation experiments or half maximal inhibition in competition experiments correspond to *Kd* values in presence of a single binding site and appropriate receptor-ligand stoichiometry, proper *Kd* calculation requires either to know the ligand and the protein concentrations (for radiolabeled ligand assays, ITC, MST), or to measure the kinetics of association and dissociation (for SPR). With insoluble chitin, PGN or uncharacterized mixtures, it is impossible to determine the molecular concentration of the ligands and thus to determine the *Kd* from saturation or competition experiments. The *Kd* values found in the literature that are deduced from half maximal incorporation or half maximal inhibition experiments have thus to be considered with caution.

Another limitation to the current biochemical characterization and understanding of LysM-RLK function is the lack of studies on specificity of the protein-ligand interaction. Where controls have been used, they are often unrelated proteins and unrelated ligands. It is thus required to use other LysM-RLK/Ps and various structures of COs, LCOs or muropeptides as controls to be able to correlate binding properties and biological functions.

### Roles of LysM-RLKs in Plant Defense Vary Between Plant Species

Plant LysM-RLK/Ps are involved in the perception of molecules that act as defense elicitors. Perception of chitin fragments relies on LYMs, LYRs and LYKs which may act in same or parallel pathways. AtLYK5 (LYRIIIC) was suggested to play a redundant role with AtLYK4 (LYRIIIA) for elicitation of defense responses through a signaling pathway depending on AtCERK1 [LYKI; (Cao et al., 2014)]. On the other hand, AtLYM2 (LYMII) was also shown to play a role in resistance to fungal pathogens. However, AtLYM2 is not required for the main ROS production in response to CO8 and has been proposed to play a role in plasmodesmata closure in response to COs. This occurs independently of AtCERK1 (Faulkner et al., 2013) raising the question of which co-receptor interacts with AtLYM2 for CO8 signaling. In rice, OsCEBIP, one of the AtLYM2 orthologs interacts with OsCERK1 (Shimizu et al., 2010) and is involved in the main ROS production in response to chitin fragments (Kaku et al., 2006). The differences found between Arabidopsis and rice for chitin perception point out the necessity of new studies on plant species in different phylogenetic clades to better understand how chitin perception has evolved in plants. Complementary roles of *AtLYK5*, *AtLYK4*, and *AtLYM2* show that genetic screens based on measurement at the plant or organ level of classical responses to MAMP (such as Ca^2+^ flux, ROS production, marker gene induction) allow to identify genes responsible for strong responses (intense and/or produced in many cells). However, such screens do not permit to find genes controlling mechanisms in specific cells that could also play important roles in pathogen resistance. This should encourage the screening of mutant collections for a variety of responses to elicitors as well as to various pathogens.

### Pathogen Effectors Target or Compete LysM-RLKs to Avoid MTI Activation

The importance of LysM-RLKs in plant defense is also highlighted by the fact that they are targeted by pathogen effectors. AtCERK1 is the target of AvrPtoB, a multi-domain and multi-function effector produced by several *P. syringae* pathovars (for review, Macho and Zipfel, 2015). AvrPtoB is known to contain an E3 ubiquitin ligase domain and to be injected in plant cells through the type III secretion system. AvrPtoB is able to ubiquitinate AtCERK1, inducing its degradation and suppressing MAMPS signaling (Gimenez-Ibanez et al., 2009). AvrPtoB is also able to interact with several AtCERK1 orthologs in tomato. Among them, SlLYK1/SlBti9 displayed a reduced kinase activity in the presence of AvrPtoB (Zeng et al., 2012). Some fungal effectors such as Avr4 and Ecp6 from *Cladosporium fulvum* suppress plant immunity in another way, by competing with plant receptor for binding to chitin fragments or interfering with receptor dimerization (for review, Sánchez-Vallet et al., 2015). For instance, Mentlak et al. (2012) showed that Ecp6 from *C. fulvum* and Slp1 from *M. oryzae* are able to bind COs with high affinity. They appear to have higher affinity than OsCEBIP for CO8 (de Jonge et al., 2010; Mentlak et al., 2012; Sánchez-Vallet et al., 2013). When added exogenously, these effectors can compete for the binding of chitin fragments to OsCEBIP (de Jonge et al., 2010; Mentlak et al., 2012). Interestingly, these effectors are composed of three LysM as the LysM-RLK/Ps. However, they might originate from an independent association of LysMs, since the loops between the LysMs are different and the highly conserved CXC motifs found in LysM-RLK/Ps are not conserved between the LysM-RLK/Ps and the LysM effectors although disulfide bridges are also involved in packing together the LysM in the effectors. The association of three LysMs in different proteins for binding chitin fragments thus represents an example of convergent evolution. Fungal effectors with LysMs are found in many fungi (Bolton et al., 2008) including pathogens with different lifestyles or host ranges suggesting that the strategy of competing for chitin binding with plant receptors in order to avoid MTI activation is widespread in fungi. Several proteins containing LysM domains were shown to play a role in fungal pathogenicity: Mg3LysM in *Mycosphaerella graminicola* (Marshall et al., 2011), ChELP1 and ChELP2 in *Colletotrichum higginsianum* (Takahara et al., 2016) and Vd2LysM in *Verticillium dahlia* (Kombrink et al., 2017).

### Functions of LysM-RLKs Involved in RNS Vary Between Legume Species

The importance of LysM-RLKs in the RNS has been unambiguously determined. Members of the phylogenetic group LYRIA and LYKI, act together for perception of Rhizobial Nod-factors. They are required for the earliest responses to LCOs, for Rhizobial colonization and for nodule development. Although demonstrated for *L. japonicus* LysM-RLKs, evidence for LCO binding to the *M. truncatula* LysM-RLKs involved in LCO perception are still lacking, questioning the similarity between these two legumes species for LCO perception. In addition, differences in sensitivity of root hairs for responses to LCOs have been detected between these species. Half of the root hairs showed calcium spiking in *M. truncatula* roots treated by up to 10^-13^ M of *Sinorhizobium meliloti* Nod-factors (Oldroyd et al., 2001; Sun et al., 2015) while no root hairs showed calcium spiking in *L. japonicus* roots treated with 10^-11^ M of *M. loti* Nod-factors (Sun et al., 2015). Most root hairs showed calcium spiking in *L. japonicus* roots treated with 10^-9^ M of *M. loti* Nod-factors (Oldroyd et al., 2001; Sun et al., 2015). Moreover, *S. meliloti*, a Rhizobial symbiont of *M. truncatula*, produces a LCO-IV(C16:2,S) as major Nod-factor (Lerouge et al., 1990) while *M. loti*, a Rhizobial symbiont of *L. japonicus*, produces LCO-V(C16:1,Cb,Fuc,Ac) as a major Nod-factor (Bek et al., 2010). A double unsaturation on the fatty acid and a sulfate groups are two LCO properties which are rare among the variety of Nod-factor structures produced by Rhizobia. Altogether, this suggests differences in LCO receptors between these two species. In contrast to CO binding proteins for which structures of their ECRs in interaction with COs have been resolved, no structural information is yet available for LCO binding proteins. Identification and characterization of the LCO binding site in LysM-RLKs will help to better understand the evolution of the LysM-RLK/P families.

### LysM-RLK/Ps Involved in Myc-Factor Perception Have Not Yet Been Identified

The role of LysM-RLK/Ps and of Myc-factors in AMS remains unclear. LCOs and short-chain COs can activate the CSSP which is essential for AMS establishment, but it is unclear whether these signals have a redundant function. Until now, two orthologous LysM-RLKs, PaNFP, and SlLYK10 have been shown to be involved in AMS establishment as AMF colonization is impaired in plants in which their expression is silenced (Op den Camp et al., 2011; Buendia et al., 2016). Because they are MtNFP/LjNFR5 orthologs, these proteins are expected to be LCO receptors although no data about their binding properties is yet published. Because these proteins are LYRs with inactive kinase, they might interact with yet unidentified LYK co-receptors. Candidates are the members of the phylogenetic group LYKI bearing the YAQ/R motif.

Other LysM-RLK/Ps are thus expected to be short-chain CO receptors and could be involved in AMS. Evidence for short-chain CO perception was already published in the 1990s. A high affinity CO4/CO5 binding site was found in a tomato cell culture (Baureithel et al., 1994). Classical responses to MAMPs such as apoplast alkalinization were found in several *Solanaceae* (including tobacco and tomato) and in rice cell cultures (Felix et al., 1999). Interestingly, CO5 concentration required to induce such response in an Arabidopsis cell culture was much higher, suggesting there is no high affinity CO4/5 binding site in this plant species. Several years after, short-chain COs (CO4 and CO5) were shown to induce calcium spiking, which is currently considered a hallmark of symbiotic responses, in various plant species establishing AMS. Moreover abundance of CO4 and CO5 in AMF exudate is stimulated by strigolactones (Genre et al., 2013), a plant hormone which is known to promote AMF colonization. It was shown that the LysM-RLK *OsCERK1* which is required for AMF colonization, is involved in CO4 perception (Carotenuto et al., 2017). OsCERK1 might interact with a high-affinity short-chain CO binding protein yet uncharacterized.

In conclusion, the role of LysM-RLKs in AMS has only started to be explored. No short-chain CO binding protein has been identified yet in any plant species. Only a few RLKs were found to be involved in AMS by the forward genetic screens or by reverse genetic approaches targeting single LysM-RLK/P performed up to now. This is likely due to the redundant functions of LysM-RLK/Ps for activation of the CSSP. Crosses to combine mutations in LysM-RLK/Ps or use of CRISPR-CAS9 technology targeting several LysM-RLK/Ps will be required to identify these genes. Considering the number of LysM-RLKs in legumes, such approaches have higher chance of success in non-legumes. However, differences between plant species in the mechanism of perception and/or in the responses to LCOs and COs as suggested by different roles of the orthologous genes *SlLYK10*/*PaNFP* and *MtNFP*/*LjNFR5*/*OsNFR5* in AMS, make this research complicated. Determining the ability of LysM-RLK/Ps to bind to short-chain COs with high affinity and reverse genetics on combinations of LysM-RLKs in various plant species will help to better understand the importance of AMF symbiotic signals for establishment of the AMS.

### Various LysM-RLKs Have Dual Roles in Symbioses and Defense

Symbiotic signals are structurally related to defense elicitors like chitin and PGN fragments. Consequently, it is logical to think that receptors should share similarities in terms of three-dimensional structure and operating mode. Several questions arise from this statement. Under an evolutionary point of view, have symbiotic receptors evolved from MAMP receptors or *vice versa*? How can a plant deal with symbiotic partners that produce both symbiotic signals and MAMPs? In the complexity of the rhizosphere microbiome, how can plants distinguish and adapt their responses when surrounded by thousands of different microorganisms? Can pathogens use the symbiotic pathway to overcome plant defense? Even if most of these questions remain unanswered, some evidence suggests crosstalk between symbiosis and defense pathways. This might occur in part through dual functions of several LysM-RLKs in symbiosis and defense pathways as shown for *OsCERK1* (essential for chitin/PGN signaling and for AMF colonization) and for *MtNFP* (essential for LCO signaling and involved in resistance to several pathogens).

Nod factors were found to transiently induce defense genes that are also induced by flg22 and chitin fragments (mix of CO2 to CO8), including PR proteins, peroxidases and transcription factors (El Yahyaoui et al., 2004; Nakagawa et al., 2011). Similarly, heterologous co-expression of two symbiotic LysM-RLKs, MtNFP and MtLYK3 or LjNFR5 and LjNFR1 induces defense responses and cell death, suggesting that these proteins can interact with signaling pathways involved in defense mechanisms (Madsen et al., 2011; Pietraszewska-Bogiel et al., 2013). Conversely, chitin fragments were found to transiently induce symbiotic genes independently of Nod-factor receptors (Nakagawa et al., 2011). Although the data were obtained through treatments with high concentration of the signal molecules and/or overexpression of the receptors, it shows possible crosstalk in the symbiosis and defense pathways.

Liang et al. (2013) demonstrated that in soybean and *A. thaliana*, perception of short-chain COs and LCOs interfere with responses induced by flg22. It is still undetermined whether the effect occurs by direct regulation of MAMP perception or signaling or because of a competition/desensitization of common actors involved in CO/LCO and MAMP signaling pathways. On the one hand, the observation of LCO effects in *A. thaliana* was surprising since this plant is not able to establish the AMS and has lost LYRI group members, known to be involved in LCO perception. On the other hand, the effect of LCO on flg22-induced responses in soybean was independent of the Nod-factor receptors suggesting that other LysM-RLK/Ps are involved in this mechanism.

An explanation to a crosstalk between symbiosis and defense pathway is that plants perceive MAMPs produced by their symbiotic partners and need to turn-off their defenses, at least locally for symbiosis establishment. Thus, it is expected that in plants able to establish the RNS and/or the AMS, symbiotic signals have a direct effect on defense mechanisms by down-regulating them. The mechanism involved in such crosstalk is, however, unknown. Up to now, the signaling pathways activated by MAMPs and the symbiotic signals appear to be different suggesting that the crosstalk between defense and symbioses occurs downstream. To better understand the role of LysM-RLK/Ps in this crosstalk, studies similar to that performed by Liang et al. (2013) have to be performed in lines mutated in LysM-RLK/Ps from species that establish the RNS and/or the AMS. Moreover, local and systemic effects of symbiotic signals on defense should be analyzed.

## Author Contributions

LB, AG, TW, and BL wrote the text. LC constructed the phylogeny trees.

## Conflict of Interest Statement

The authors declare that the research was conducted in the absence of any commercial or financial relationships that could be construed as a potential conflict of interest.

## Footnotes

1 http://gpcr.biocomp.unibo.it/predgpi/pred.htm

2 http://labtools.ipkgatersleben

## Abbreviations

AA: amino acids
AMF: arbuscular mycorrhizal fungi
AMS: arbuscular mycorrhizal symbiosis
CO: chitooligosaccharide
CSSP: common symbiosis signaling pathway
ECR: extracellular region
EPS: exopolysaccharide
ER: endoplasmic reticulum
ETI: effector-triggered immunity
GlcNAc: *N*-acetyl glucosamine
GPI: glycosylphosphatidylinositol
ICR: intracellular region
IT: infection thread
ITC: isothermal titration calorimetry
LCO: lipo-chitooligosaccharide
LPS: lipopolysaccharide
LRR: leucine rich repeat
LYK: LysM domain–containing receptor-like kinases
LYM: LysM-proteins
LYR: LYK-related
LysM: lysin motif
MAMP: microbe-associated molecular pattern
MST: microscale thermophoresis
MTI: MAMP-triggered immunity
NMR: nuclear magnetic resonance
PGN: peptidoglycan
PM: plasma membrane
RLK: receptor-like kinase
RLP: receptor-like protein
RNAi: RNA interference
RNS: root nodule symbiosis
ROS: reactive oxygen species
SP: signal peptide
TM: transmembrane domain
VIGS: virus induced gene silencing
WT: wild type
