# Supplementary material for: LysM Receptor-Like Kinase and LysM Receptor-Like Protein Families: An Update on Phylogeny and Functional Characterization
Source: Front Plant Sci. 2018 Oct 24;9:1531. doi: 10.3389/fpls.2018.01531 (PMC6207691; doi:10.3389/fpls.2018.01531)
Supplement: Supplementary file 1 [file Data_Sheet_1.PDF]

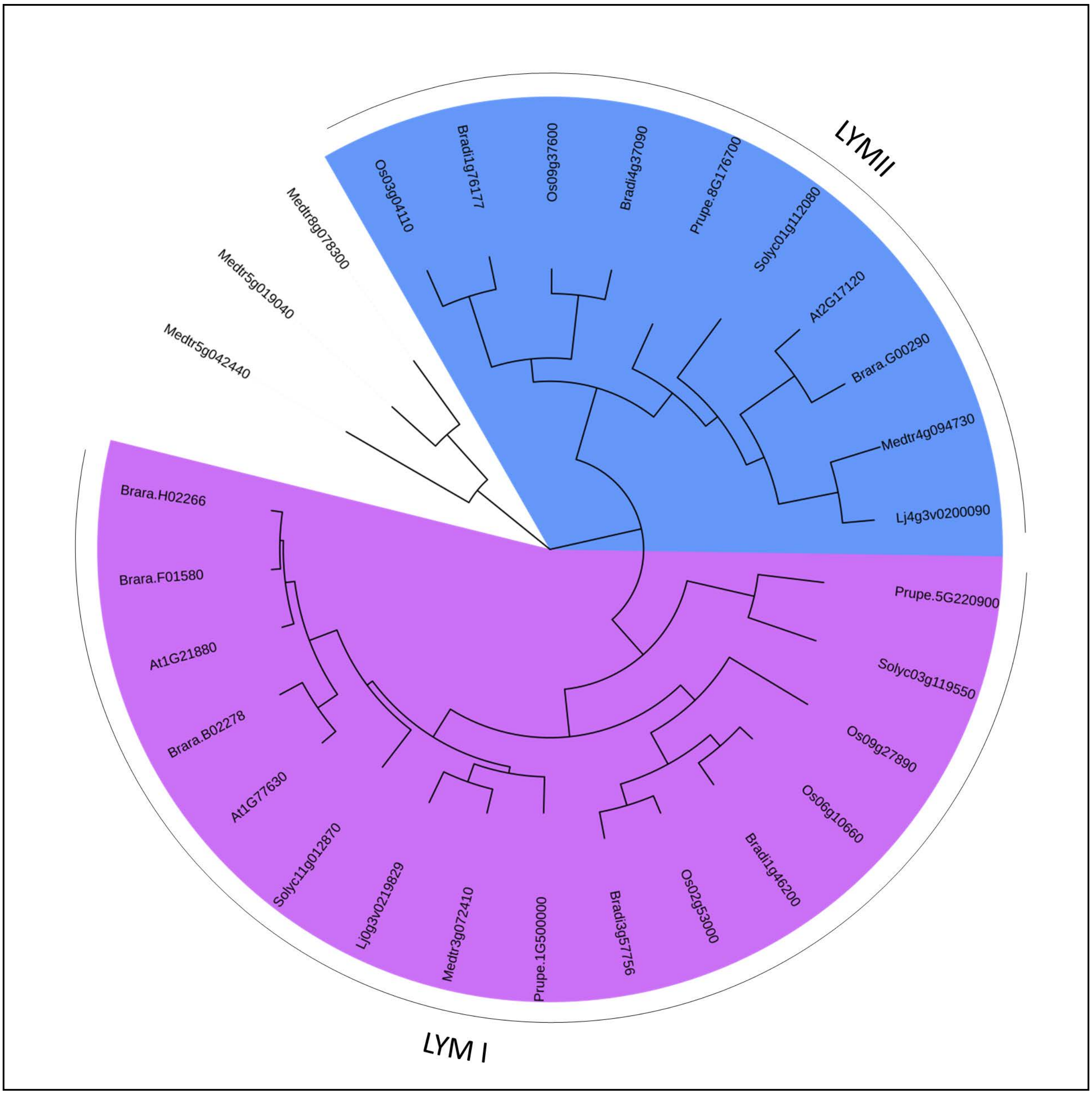


**Fig. S1: MrBayes phylogenetic tree of the LYMs.** Different phylogenetic groups are shown in different colors. ECR of 3 LYR proteins were used as outgroup sequences. Protein sequence alignment was produced as in Fig. 3. The tree was computed thanks to a bayesian method with MrBayes version 3.2.5 (Huelsenbeck & Ronquist, 2001; Ronquist *et al.*, 2012 ; parameters: ngen= 400000, samplefreq=100, relburnin=yes, burninfrac= 0.25).

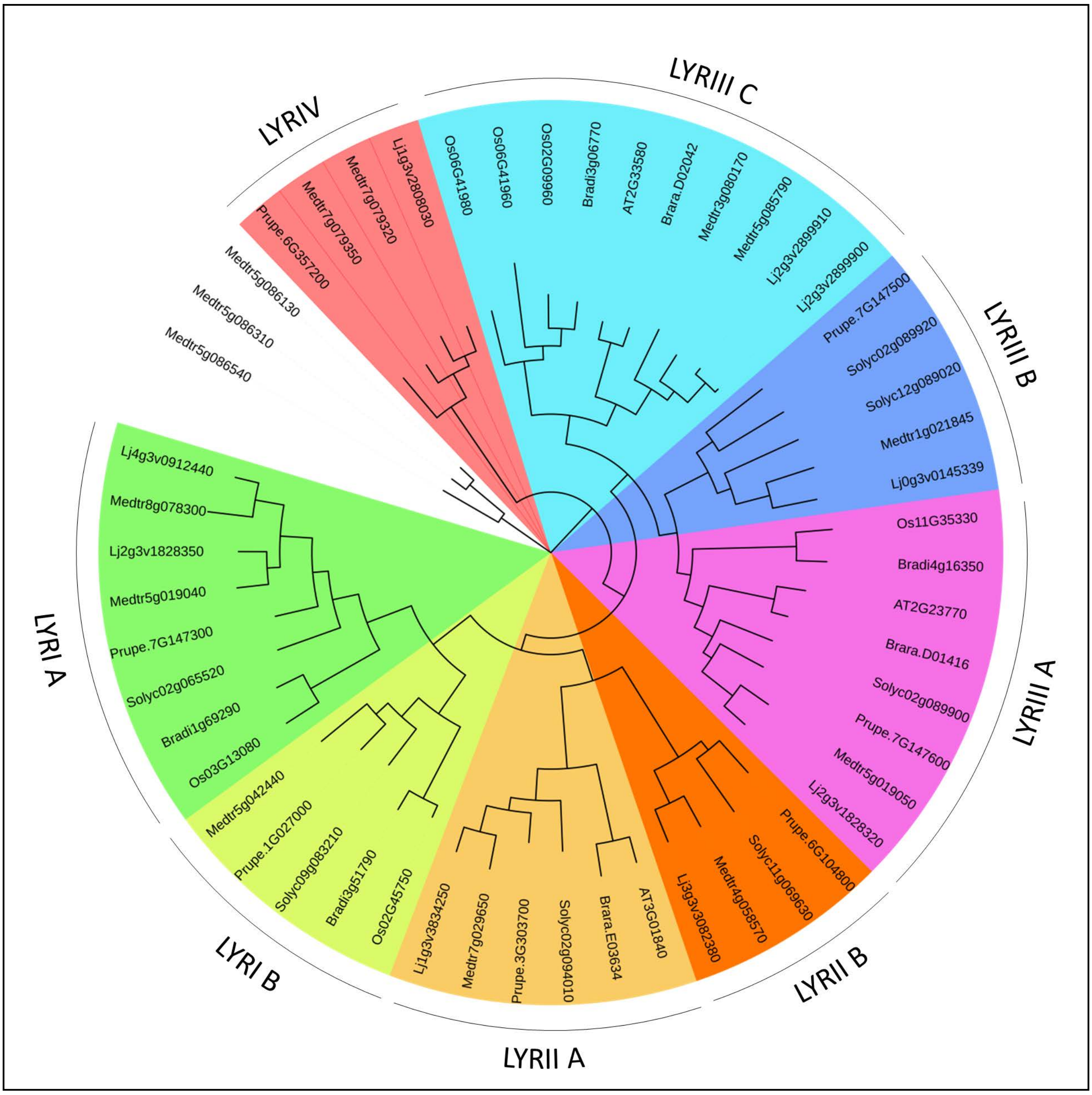


**Fig. S2: MrBayes phylogenetic tree of the LYRs.** Different phylogenetic groups are shown in different colors. ECR of 3 LYK proteins were used as outgroup sequences. Tree was built as in Fig. S1.

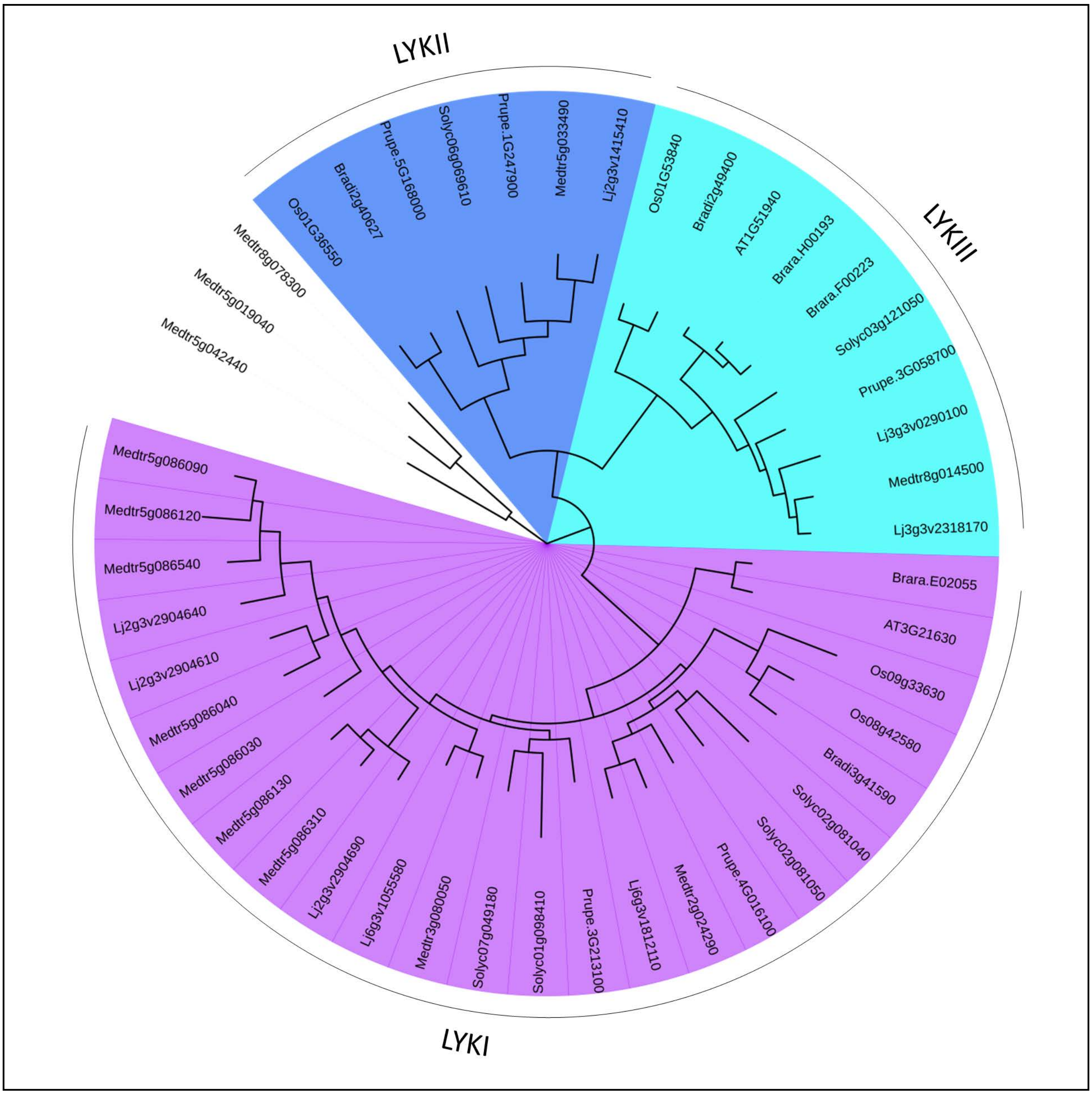


**Fig. S3: MrBayes phylogenetic tree of the LYKs.** Different phylogenetic groups are shown in different colors. ECR of 3 LYR proteins were used as outgroup sequences. Tree was built as in Fig. S1.

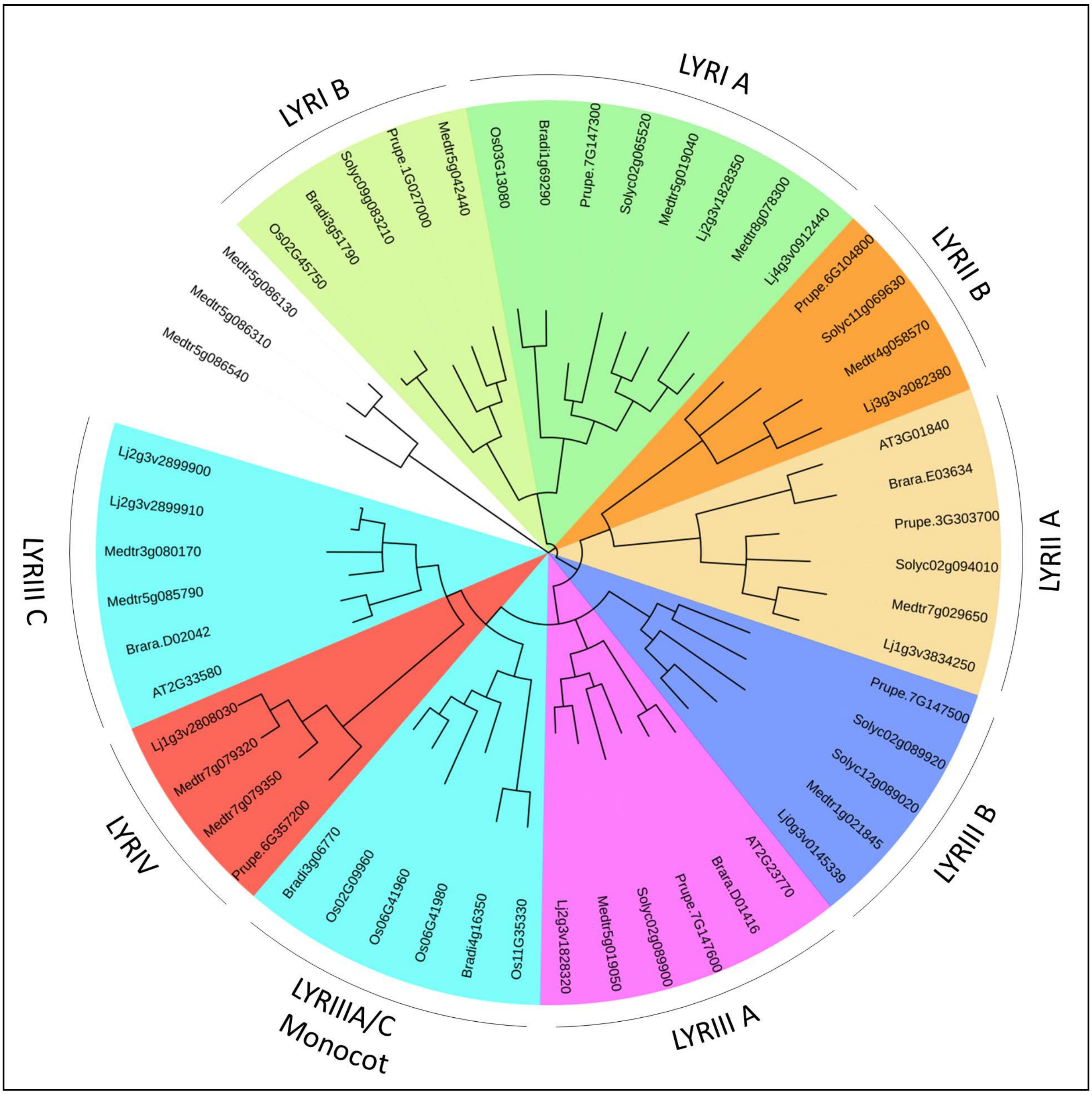


**Fig. S4: MrBayes phylogenetic tree of the LYRs using Gblock.** Different phylogenetic groups are shown in different colors. ECR of 3 LYK proteins were used as outgroup sequences. Tree was built as in Fig. S1.

## References:

Huelsenbeck JP, Ronquist F. 2001. MRBAYES: Bayesian inference of phylogenetic trees. *Bioinformatics* 17: 754-755.

Ronquist F, Teslenko M, van der Mark P, Ayres DL, Darling A, Höhna S, Larget B, Liu L, Suchard MA, Huelsenbeck JP. 2012. MrBayes 3.2: efficient Bayesian phylogenetic inference and model choice across a large model space. *Syst Biol* 61: 539-542.
